# High niche specificity and host genetic diversity of groundwater viruses

**DOI:** 10.1093/ismejo/wrae035

**Published:** 2024-03-07

**Authors:** Emilie Gios, Olivia E Mosley, Michael Hoggard, Kim M Handley

**Affiliations:** School of Biological Sciences, The University of Auckland, Auckland 1010, New Zealand; NINA, Norwegian Institute for Nature Research, Trondheim 7034, Norway; School of Biological Sciences, The University of Auckland, Auckland 1010, New Zealand; NatureMetrics Ltd, Surrey Research Park, Guildford GU2 7HJ, United Kingdom; School of Biological Sciences, The University of Auckland, Auckland 1010, New Zealand; School of Biological Sciences, The University of Auckland, Auckland 1010, New Zealand

**Keywords:** bacteriophage, virus, auxiliary metabolic gene, biogeochemical cycles, groundwater, aquifer

## Abstract

Viruses are key members of microbial communities that exert control over host abundance and metabolism, thereby influencing ecosystem processes and biogeochemical cycles. Aquifers are known to host taxonomically diverse microbial life, yet little is known about viruses infecting groundwater microbial communities. Here, we analysed 16 metagenomes from a broad range of groundwater physicochemistries. We recovered 1571 viral genomes that clustered into 468 high-quality viral operational taxonomic units. At least 15% were observed to be transcriptionally active, although lysis was likely constrained by the resource-limited groundwater environment. Most were unclassified (95%), and the remaining 5% were *Caudoviricetes*. Comparisons with viruses inhabiting other aquifers revealed no shared species, indicating substantial unexplored viral diversity. *In silico* predictions linked 22.4% of the viruses to microbial host populations, including to ultra-small prokaryotes, such as *Patescibacteria* and *Nanoarchaeota*. Many predicted hosts were associated with the biogeochemical cycling of carbon, nitrogen, and sulfur. Metabolic predictions revealed the presence of 205 putative auxiliary metabolic genes, involved in diverse processes associated with the utilization of the host’s intracellular resources for biosynthesis and transformation reactions, including those involved in nucleotide sugar, glycan, cofactor, and vitamin metabolism. Viruses, prokaryotes overall, and predicted prokaryotic hosts exhibited narrow spatial distributions, and relative abundance correlations with the same groundwater parameters (e.g. dissolved oxygen, nitrate, and iron), consistent with host control over viral distributions. Results provide insights into underexplored groundwater viruses, and indicate the large extent to which viruses may manipulate microbial communities and biogeochemistry in the terrestrial subsurface.

## Introduction

Viruses are thought to infect all cellular forms of life, including bacteria (as “bacteriophage” or “phage”) and archaea. Phage are known to shape the structure and functioning of microbial communities, and thus microbial processes and networks in the environment, by infection and lysis of host cells [1]. Their influence on microbial communities has been extensively studied in oceanic systems, where it has been estimated that between 20% and 40% of all marine bacterial cells are lysed each day as a consequence of viral infection [2]. In these environments, the biogeochemical cycling of elements such as carbon, nitrogen, and sulfur is also influenced by virus-mediated control of species composition, microbial activity, and evolutionary trajectories [1, 3].

Numerous studies have shown that viruses can acquire genes, termed auxiliary metabolic genes (AMGs), from their host through lateral transfer of genetic material [4]. Through their expression, these host-derived genes have been shown to “hijack” or supplement host metabolism (particularly central metabolic pathways) [5, 6], and to participate in ecosystem functioning by encoding enzymes involved in biogeochemical transformations. By imparting new functions they can also expand host environmental niches [4]. Examples of AMGs include genes involved in photosynthesis, which were among the first AMGs described [5, 7-9]; nucleotide metabolism [5, 10]; the cycling of nitrogen [11], phosphorus [12], and sulfur [11, 13]; and diverse carbon metabolisms, including methane oxidation [14].

To date, only a small number of studies have explored the ecology of viral communities in groundwater ecosystems [15-19], demonstrating that the abundance of viral particles exceeds that of prokaryotic cells, and that viral diversity is niche-specific. Screening of viral particles in groundwater has mainly been employed as an indicator of drinking water quality. As such, not much is known about the characteristics and diversity of resident viral communities of aquifers. However, because subsurface environments such as aquifers are largely dominated by prokaryotic life [20], viral communities in these environments are likely to also be dominated by bacteria and archaea viruses, and to a lesser extent viruses associated with microeukaryotes and stygofauna. The extraordinarily high prokaryotic richness and phylogenetic diversity in terrestrial aquifers, which includes divergent bacterial and archaeal groups (e.g. Candidate Phyla Radiation [CPR] and DPANN archaea) [21, 22], suggests they are an abundant reservoir of unexplored viral species and metabolic diversity.

To assess the diversity, environmental distributions, and potential ecosystem role of viruses in the terrestrial subsurface, we analysed 16 metagenomes and six metatranscriptomes from two aquifers, and recovered hundreds of novel groundwater viral genomes. *In silico* methods were employed to identify putative hosts for a significant fraction of the recovered viruses. Associations between viruses and groundwater physicochemistries were assessed to explore viral niches, and how environmental factors influence host-virus relationships. Finally, we evaluated the potential for groundwater viruses to shape microbial processes through the expression of AMGs. Our findings contribute hundreds of novel viruses to the rapidly expanding inventory of viral genomes, along with their host-derived metabolic genes, and provide insights into viral diversity and ecosystem function in aquifers.

## Materials and methods

### Sample collection and omic data generation

Groundwater samples, targeting the ≥0.22 μm prokaryotic and associated intracellular viral fraction, were collected from two aquifers in Canterbury, New Zealand, and metagenomes and metatranscriptomes generated, along with metagenome assemblies and determination of metagenome-assembled genomes (MAGs), as described previously [23]. Sixteen metagenomes (samples gwj01-gwj16) were generated from eight groundwater wells (per well, one filtered groundwater sample before [odd sample numbers] and after sonication [even sample numbers] to enrich biomass [sediment particles and biofilms]). Six metatranscriptomes were generated from four of these samples (four before sonication [samples gwj09, gwj11, gwj13, and gwj15], and two after sonication [samples gwj14 and gwj16]). De novo assembled scaffolds from the metagenomes were used to identify viral genomes (see below). Analysis of prospective hosts included 396 medium to high quality dereplicated prokaryotic MAGs (≥70% completeness, <5% contamination, 99% average nucleotide identity (ANI) dereplication threshold) [23].

### Groundwater geochemical measurements

Methods for measurement of the following groundwater parameters are described previously [23, 24]: dissolved oxygen (DO), dissolved organic carbon (DOC), dissolved reactive phosphorus (DRP), nitrate-N, redox potential (ORP), pH, specific conductivity (SPC), sulfate, and water temperature. Additionally, total phosphorus and phosphate were determined according to American Public Health Association (APHA) 4500-P B & E (modified to include an acidic ammonium persulfate to convert organophosphates and polyphosphates to orthophosphate) [25]. Total alkalinity was analysed according to APHA 2320 B [25]. Total calcium, iron, magnesium, potassium, and sodium were measured according to APHA 3125 B [25]. Total suspended solids were analysed according to APHA 2540 D, and chloride according to APHA 4110 B [25].

### Prokaryotic community analysis

Taxonomic classification of MAGs used GTDB-Tk v2.1.1 [26] (classify_wf default workflow) with database release 124. Phylogenetic trees were built using 120 bacterial and 122 archaeal concatenated marker gene alignments generated by GTDB-Tk and FastTree v2.1.11 [27] (JTT + CAT model over 1000 bootstraps). The resulting trees were visualized and annotated in iTOL v6 [28]. Metabolic pathways were determined using KEGGDecoder v1.1 [29], which parses annotations generated with KofamKOALA (*e*-value ≤ 0.001) [30].

### Identification of viral contigs

Viral sequences were identified from metagenome assemblies using a combination of VirSorter2 v2.1 [31], VIBRANT v1.2.1 [32], and DeepVirFinder v1.0 [33]. Tools were run with the default settings, with the following exceptions: contigs were retained if ≥5 kb and (i) score >0.9 for VirSorter2; (ii) all contigs based on VIBRANT default settings; (iii) score >0.9 and *P* <0.05 for DeepVirfinder. Putative eukaryotic contigs were identified from DeepVirFinder contigs using Kraken v2.1.1 [34] and removed, as this software may identify eukaryotic sequences as viral [33]. Filtered contigs identified with all methods across all metagenomic assemblies were dereplicated using the BBMap v38.81 dedupe.sh script (https://sourceforge.net/projects/bbmap/; parameters: minidentity = 100, exact = f).

Viral contigs were clustered into viral OTUs (vOTUs) based on ≥95% nucleotide identity over ≥85% coverage of the shorter sequence [35], using the Cluster_genomes_5.1.pl script with MUMMER v4.0.0b2 [36] (https://github.com/simroux/ClusterGenomes; parameters: -c 85 -i 95). The longest sequence of the vOTU cluster was retained as the representative sequence, and vOTUs retained if ≥10 kb. The quality of the viral sequences was assessed using CheckV v0.7.0 [37] (end_to_end), and only high quality vOTUs (based on MIUViG quality criteria [35]) containing at least one viral gene were retained. A final filtering step was performed to remove viral contigs where a significant portion of genes were predicted to be host genes, using the following criteria: viral gene count = 0; proportion of host genes >30% of total vOTU length; and ratio host genes:viral genes >10:1. This removed a further 15 vOTUs, mostly identified by DeepVirFinder.

### Relative abundance profiles of prokaryotic MAGs and vOTUs

To determine relative abundance profiles, quality trimmed metagenomic reads were mapped onto dereplicated MAGs and the vOTU sequences using Bowtie v1.2.0 (−n 1 -l 222 --minins 200 --maxins 800 -best) [38]. MAG and vOTU read mapping was conducted independently. MAG coverages were determined by dividing summed mapped read lengths by summed contig lengths, and vOTU read coverages were divided by contig length. Sample-specific MAG and vOTU coverages were normalized by library size, as described previously [39], facilitating coverage comparisons across samples.

### Taxonomic classification of vOTUs

For each viral genome, protein coding sequences were predicted using prodigal v2.6.3 (−p meta) [40]. The resulting sequences were then used as input to build a genome network based on shared protein content using vConTACT v2.0 [41]. NCBI “ProkaryoticViralRefSeq201-Merged” database was selected as the reference database. Due to recent restructuring of DNA virus family, genus, and species taxonomy in the viral NCBI RefSeq, references were updated based on the latest RefSeq metadata (accessed 16 September 2023), with higher taxonomic ranks propagated by matching to the ICTV species list (MSL38.v3; https://ictv.global/taxonomy; accessed 16 September 2023). Within vConTACT v2.0, Diamond v2.0.6 [42] was used for protein–protein comparison, MCL v14.137 [43] for protein clustering, and ClusterOne v1.0 [44] for genome clustering. Genus-level taxonomic predictions were made based on viral clusters generated by vConTACT v2.0 [41]. The resulting network was visualized using Cytoscape v3.8.2 [45].

### Nucleotide and protein clustering with IMG/VR

We used rapid genome clustering based on pairwise ANI to compare vOTU sequences recovered in this study to known viruses using anicalc.py and aniclust.py, distributed as part of the CheckV package (https://bitbucket.org/berkeleylab/checkv/src/master/). For this, vOTUs were compared to all viruses in the IMG/VR v3 database (downloaded on 12 May 2021) and groundwater viruses in the larger v4 database (1 December 2024). Viral OTUs were also compared with groundwater viruses from the IMG/VR v4 database via protein clustering in vConTACT2 as per the methods outlined above.

### Virus–host predictions

Viral OTU representatives were linked to putative prokaryotic host genomes using a combination of four parameters with the following ranked criteria (most to least robust) [12, 46]: (i) CRISPR linkage; (ii and iii) tRNA or nucleotide sequence homology; (iv) oligonucleotide frequency (ONF) similarity. Individual tool parameters were as follows. (i) CRISPR spacer homology: CRISPR spacers and repeat elements were assembled from quality filtered metagenomic reads using Crass v1.0.1 [47] with default parameters. Recovered CRISPR spacer sequences were compared to viral and prokaryotic contigs respectively using BLASTn. Viruses and prokaryotes that matched the same CRISPR spacer with ≤1 mismatch over the full length of the spacer were considered linked. Further confirmation of functional CRISPR-Cas systems in the prokaryote MAGs was assessed via CRISPRCasFinder v4.2.30 [48] with the optional parameters -def G -ms 20 -xs 75. Following identification of putative CRISPR repeat regions, CRISPRCasFinder searches for associated CAS genes within the same genomic region. This process is likely to be very conservative in fragmented MAG genomes due to CRISPR-Cas systems potentially being split across separate assembled contigs, but was included as further evidence for high-confidence viral-host matches identified via CRISPR spacer homology. (ii) Transfer RNA (tRNA) homology: tRNAs were identified from vOTUs and prokaryotic MAGs using ARAGORN v1.2.38 (−t -gcstd except for *Gracilibacteria*: -t -gcgrac) [49]. Viral tRNA sequences were compared to prokaryotic sequences using BLASTn, and match requirements were set as ≥90% nucleotide identity over ≥90% of the query sequence length. (iii) Nucleotide sequence homology: Viral and prokaryotic contigs were compared using BLASTn. Host-virus linkage was predicted if matches fulfilled the following criteria: *e*-value ≤0.001, ≥70% nucleotide identity, >70% query (vOTU) coverage (minimum query length 10 kb), ≥50 bit-score. (iv) Oligonucleotide frequency (ONF): VirHostMatcher v1.0.0 [50] was used to compute ONF based distances between viral and host genomic populations using default parameters. Only VirHostMatcher matches with d2^*^ values ≤0.2 were retained. Finally, taking all matches together, where multiple hosts were predicted for a given vOTU the most probable host was selected using the following criteria in order of priority: (i) the linkage supported by the most approaches, and (ii) for linkages supported by an equal number of tools, the combination with the most robust methods (as indicated above) was selected. Additionally, for multiple hosts predicted by VirHostMatcher, the one with the lowest score (highest match similarity) was selected as the most probable host. Most probable hosts were selected for all vOTUs, excluding five with multi-host matches predicted by tRNAs.

### Identification of auxiliary metabolic genes (AMGs)

Gene annotations and putative AMGs were predicted for all vOTUs using DRAM-v v1.3.5 [51] and a modified version VIBRANT. VIBRANT was modified to omit any filtering steps to solely generate annotations for all contigs (see Supplementary Materials). For DRAM-v, the options --remove_transposons and --remove_fs were included to exclude genes on scaffolds containing a transposon or those near the ends of scaffolds as potential AMGs. AMGs predicted by DRAM-v were filtered based on the following criteria: auxiliary_score ≤3 && amg_flag M NOT T|F|V|A|P|B. In addition, predictions were manually inspected to ensure that AMGs were not host-contamination: each AMG was required to be preceded and followed by known viral genes (at least assigned to a viral orthologous group [VOG] or to a known viral PFAM domain). AMG predictions from DRAM-v and VIBRANT were cross-matched, and AMGs predicted with both tools were considered as “high” confidence AMGs, whereas AMGs predicted with DRAM-v only were considered as “low” confidence. In cases where vOTUs were putatively matched with hosts, AMGs were compared to host genomes using BLASTp to assess the relatedness of identified AMGs to homologous genes found in the host.

### Gene expression analysis

For transcriptomic analyses, adapter sequences were removed using cutadapt v2.10 (https://github.com/marcelm/cutadapt). Reads were then further quality trimmed using sickle v01.33 (parameters -q 30 -l 80; https://github.com/najoshi/sickle), and residual rRNA sequences were removed using SortMeRNA v2.1 [52]. Filtered transcriptomic reads were mapped to predicted gene sequences from the set of vOTUs using Bowtie2 v2.3.5 (--end-to-end --very_sensitive) [53]. Read counts were determined using featureCounts v1.6.3 (-F SAF) [54]. Singleton reads per gene were removed, and the remaining read counts were normalized to a modified version of transcripts per kilobase per million reads mapped (TPM) using the following equation: (number of reads mapped to gene)^*^(1000/gene length)^*^(1 000 000/library size).

### Presence/absence and statistical analysis

MAGs and vOTUs were deemed active, or present in a sample, if mapped WTS, or WGS, read length was >10% of genome length. All statistical analyses and data visualizations were performed in R v4.0.2 [55], unless specified otherwise. Alpha diversity (richness and Shannon Index), beta diversity (Bray–Curtis dissimilarities), and distance-based redundancy analysis (dbRDA) (based on Bray–Curtis dissimilarities) were calculated for prokaryote and viral community fractions using the R “vegan” package (v2.5-6) [56]. To assess the relationships between the abundances of viruses, their hosts, and environmental parameters, Pearson correlations were performed between the normalized coverage of each genome (viral or prokaryotic) and each groundwater variable using the R base “stats” package. Results were examined to verify whether members of host/virus pairs correlated to the same variable.

## Results and discussion

### Groundwater viruses recovered

**Figure 1:**
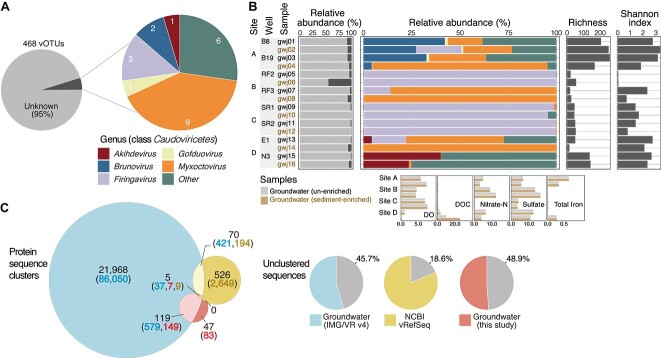
Abundance and diversity of groundwater viral communities. (A) Predicted taxonomy of groundwater vOTUs based on protein clustering with viral sequences from NCBI vRefSeq sequences v201. (B) Relative abundance of unclassified vOTUs (top left); relative abundance of taxonomically classified viruses only (top middle); richness and Shannon index based on the total groundwater vOTU representative sequences (top right). Groundwater chemistry (bottom left, units mg/l for DO, and g/m^3^ for other analytes). (C) Summary of protein sharing network (Fig. S3) between high-quality groundwater vOTUs in this study (*n* = 468), NCBI vRefSeq sequences v201 (*n* = 3502), and all groundwater viral sequences from IMG/VR v4 (*n* = 160 465). In the Venn diagram, numbers of viral clusters obtained using vConTACT2 are given in black font. Numbers of genomes in clusters are given in parentheses, including those shared from each dataset as shown in the Venn diagram overlaps (blue-filled font = groundwater IMG/VR; yellow-filled font = NCBI vRefSeq; red-filled font = groundwater this study). Pie charts show the proportions of unclustered viral genomes per dataset represented by gray shading. Sequences considered as unclustered were characterized by vConTACT2 as “outliers”, “singletons”, “overlap” cluster status. Numbers of unclustered genomes for each dataset: Groundwater IMG/VR (*n* = 73 378), NCBI vRefSeq (*n* = 650), groundwater vOTUs in this study (*n* = 229).

A total of 247 666 viral contigs were identified across all 16 groundwater samples. The majority of recovered viral genomes likely belonged to viruses actively infecting bacteria or archaea (i.e. the intracellular fraction), given the aquifer environments were dominated by prokaryotic communities (between 85 and 89% of all metagenomic reads were assigned to bacteria and archaea using MEGAN; Table S1 and Supplementary Materials), and the sampling filter size used was suitable for collecting microbial cells (0.22 μm). Although it is also likely that sampled viruses included viral particles, such as those unable to pass through blocked filter pores, or those sorbed to sediment particles [57]. Indeed, sediment-enriched samples contained viral communities with broadly similar composition (based on available genus level classifications), richness, and diversity as unenriched groundwater collected directly beforehand from the same groundwater wells (Fig. 1A). Viral sequences were clustered at 95% ANI into 5672 vOTUs (see Fig. S1 for vOTU selection by method), representing a species-level taxonomy as previously proposed for uncultivated virus genomes [35]. These included 468 high-quality complete or near-complete (90%–100%) vOTU genome sequences ≥10 kb long, representing 1571 clustered viral contigs.

On average, viral genomes were 50 271 ± 35 812 bp long (± standard deviation, SD) (Fig. S2, Table S2), which is a typical genome size range based on the sequence lengths observed in the NCBI Viral RefSeq v205 database (60% of genomes in the database were <100 kb as of 14 May 2021). Five vOTUs were identified with representative sequences ≥200 kb in length (2.2–3.8 Mbp, Table S2), and thus could be “jumbo” phage [58]. Of these, two carried tubulin-like protein-coding genes, which are involved in the assembly of a nucleus-like structure that encapsulates jumbo phage DNA within the host cell during infection [59].

### Taxonomy and diversity of groundwater viruses

Taxonomic prediction of high-quality vOTUs was performed using genome-based network analysis of their shared protein content against known viruses from NCBI RefSeq database v201 (with taxonomy updated based on the latest NCBI RefSeq metadata, accessed 16 September 2023). Of these, 95% of vOTUs remained unclassified based on vConTACT2 clustering thresholds (Fig. 1A), highlighting the considerable viral novelty harbored by these understudied ecosystems. The remaining viruses were assigned to the *Caudoviricetes* class, specifically the *Myxoctovirus* (*n* = 9), *Firingavirus* (*n* = 3), *Brunovirus* (*n* = 2), and other genera (*n* = 8). *Caudoviricetes* are dsDNA tailed phages, a morphology that characterizes the vast majority of viruses infecting bacteria and archaea [60, 61]. They appear to be widespread in aquatic environments [62-65], including ones identified in aquifers by previous genomic [15-17, 66] and microscopy [67, 68] studies. Based on relative abundance profiles of the 468 high quality vOTUs recovered, between 55.1% and 99.6% of the total viral abundance per sample was attributed to unclassified viruses (Fig. 1B).

To place groundwater viral populations in a broader context, we determined the similarity of our viral dataset to viruses from other ecosystems. Groundwater viral genomes were compared at the vOTU level using ANI clustering to the IMG/VR v3 database (all samples, *n* = 2 377 994), which contains viral sequences from a wide range of environments (e.g. aquatic, terrestrial, host-associated). Only two vOTUs from our study were found to cluster with all IMG/VR v3 viral sequences. This highlights that most groundwater viruses are not just taxonomically unique, but also unique compared to those in other ecosystems, and suggests almost all viral genomes analysed here are from novel species [35]. Furthermore, when clustering against only groundwater samples in the IMG/VR v4 database (*n* = 160 465), none clustered with other groundwater viral populations based on a 95% ANI similarity threshold, despite general commonalities in groundwater prokaryote compositions [24]. At the protein level, however, similarity with previously reported groundwater viruses was observed (Fig. 1C, Fig. S3). A protein sharing network revealed 124 (27%) of our high-quality groundwater virus genomes clustered with those from aquifers globally, suggesting that many viruses found in these habitats globally share at least genus-level relatedness.

**Figure 2:**
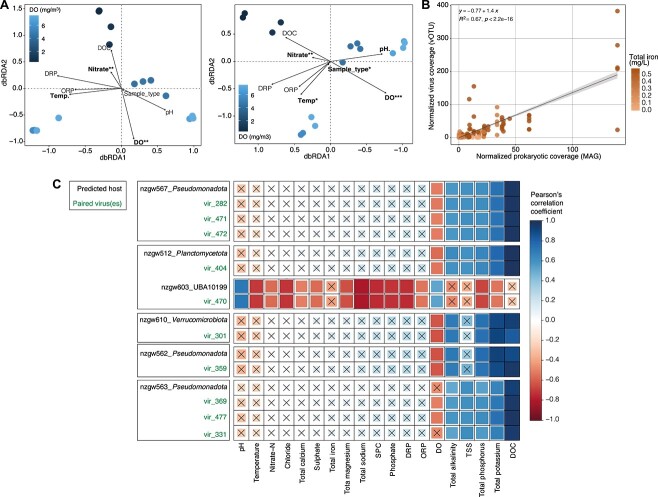
Impact of groundwater parameters on total viral communities and host-virus relationship. (A) Distance-based redundancy analysis (dbRDA) of bray-Curtis dissimilarities between 16 groundwater viral communities based on normalized coverage data (viral community fraction only, left plot), and 16 groundwater prokaryotic communities based on normalized coverage data (prokaryotic community fraction only, right plot). Samples are colored according to groundwater dissolved oxygen (DO) concentrations (mg/m^3^). The vectors indicate fitted environmental variables significantly correlated to dbRDA coordinates (permutation test, permutations = 999; ^.^*P* <0.1, ^*^P<0.05, ^*^^*^P <0.01, ^*^^*^^*^P <0.001). Percentage of variance explained: 24.51 (dbRDA1) and 19.16 (dbRDA2). (B) Normalized coverages of each of the vOTU-host pairs that also showed significant (*P* <0.05) Pearson’s correlation to groundwater total iron content. The regression line was placed based on fitting the host *versus* virus pair abundance data to a linear model, and the shaded area represents 95% confidence interval. Data points are additionally colored by sample total iron content. Percentage of variance explained: 22.73 (dbRDA1) and 19.67 (dbRDA2). (C) Example of Pearson’s correlation coefficients (for top 10 pairs exhibiting the most consistent correlations with groundwater physicochemistries; Table S8) for host lineages and their viruses (one to three per host), correlated with environmental and geochemical measurements (significant when *P* <0.05; non-significant correlations are depicted with an “x”).

Species richness calculations based on viral contigs showed that between 15 and 258 high quality vOTUs were present per sample (Fig. 1B, Table S3), and unlike read annotation alone, viral genome assembly enabled detection of viruses across all samples (Table S1 versus Table S4). Both richness and Shannon index varied with sampling locations, and were highest in samples with higher groundwater iron contents (see discussion below on correlation between viral abundance and iron concentrations). Viral community alpha diversity did not follow that of prokaryotic communities, which were comparatively invariant across groundwater samples (Table S3). However, based on the recruitment of metagenomic sequencing reads, only 11 of the 468 vOTUs were detected in at least 50% of samples (Table S4), indicating a restricted distribution in groundwater. This is consistent with the site-specific diversity observed for the corresponding prokaryote populations (Fig. 2, Table S5) [69], and as shown for the substantial ultra-small prokaryote fraction (CPR and DPANN archaea) in these samples and groundwater elsewhere [22, 24].

### Virus-host linkages in groundwater

To examine the potential impact of the recovered viruses on cohabiting prokaryotic communities, we linked vOTUs to putative hosts *in silico*. We screened the 396 groundwater bacterial and archaeal MAGs, recovered from the same metagenomic dataset, for genomic features linking hosts with bacteriophage and archaeal viruses. Putative hosts were predicted for 105 (22.4%) of the 468 groundwater vOTUs (Table S6). This is a similar fraction of viruses linked to hosts as found in other environments using similar methods [46, 70].

**Figure 3:**
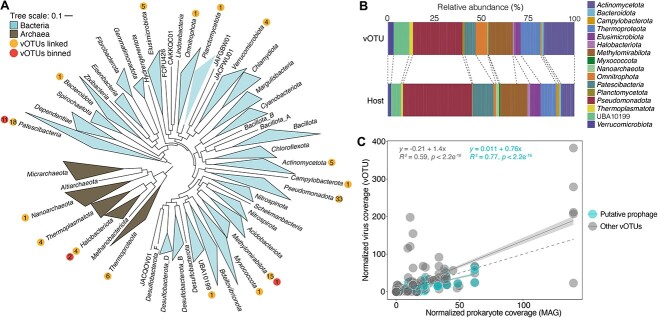
Host-virus linkages and relative abundance patterns. (A) Phylogenetic trees of the recovered bacterial (unrooted) and archaeal (rooted at midpoint) MAGs. Trees are based on either 120 concatenated bacterial marker genes or 122 concatenated archaeal marker genes from GTDB-Tk. Scale bar indicates the number of substitutions per site. The number of viruses infecting members of each lineage are indicated as numbers in orange circles (inner bubble). The red circled numbers indicate the number of viral genomes binned within a predicted host genome (outer bubble). (B) Overall relative abundance profiles (summed across all samples) of groundwater vOTUs and their putative hosts grouped by the host taxonomy at phylum level. (C) Significant Pearson’s correlation between relative abundances of vOTUs and their putative hosts. Regression line was placed based on fitting data to a linear model (gray shading = 95% confidence interval), and dashed line indicates 1:1 correlation.

Predicted groundwater hosts comprised diverse prokaryotes, spanning 29 bacterial and archaeal phyla (132 MAGs in total; Fig. 3, Table S6). Of these, 14 viruses were co-binned with bacterial or archaeal MAGs, and six were linked via CRISPR spacers. On average there were 11.8 ± 17.8 predicted hosts per virus, and 2.4 ± 2.6 viruses per host. Virus specificity and host ranges can vary greatly, from species- or strain-specific viruses [71] to broad-host-range (generalist) phage [72-74]. Additionally, host cells can be co-infected by multiple different viruses, as has been described previously in marine bacteria [75]. Here, just over half (54 [51.5%]) of groundwater vOTUs with predicted hosts were linked to members of a single prokaryotic phylum, with the majority (41 vOTUs) predicted to be specialists associated with a single prokaryotic MAG (Table S6), although some hosts may not have been detected. The other half of viruses were linked to hosts ranging across diverse phyla, of which most were domain specific (35 vOTUs), although some were distributed across both bacterial and archaeal domains (16 vOTUs; Table S6). Most predicted hosts spanning multiple phyla were predicted by oligonucleotide frequency similarities, and are hence speculative, although two were predicted with high confidence by three separate methods (CRISPR/tRNAs, oligonucleotide frequencies, and nucleotide homology for vir_28—*Thermoproteota*/*Halobacteriota* and vir_470—UBA10199/*Bacteroidota*; Table S6).

Viruses with wide host ranges infect hosts across genera [72], orders and classes [73, 76, 77], phyla [78], or across domains [74]. Host specificity relies on several mechanisms occurring at every step of the phage life cycle, including surface adhesion to the host membrane via receptor binding proteins [79], adaptations to the molecular mechanisms of the host, and intracellular defense systems [80, 81]. Metagenome-based predictions from a large range of other habitats [10, 80, 81] have illustrated a tendency towards habitat and host specificity (e.g. infecting closely related hosts), alongside the potential broad host ranges of some viruses. Results of host range prediction here (using similar criteria as used previously [11]—mostly oligonucleotide frequencies) likewise exhibited a bias among viruses towards higher host specificity (Fig. S4). Despite this, a greater tendency towards generalism was predicted among groundwater phage than reported in these other studies (i.e. 51 vOTUs with hosts predicted to span multiple phyla), and may reflect differences in the environmental conditions (e.g. aquifers are characterized by scarce organic carbon and nutrients, low biomass, low flow rates, and high spatial heterogeneity [24]) or methodology (notably oligonucleotide frequency prediction). Nonetheless, the high confidence multi-phyla host matches for vir_28 and vir_470 suggest that potential generalism in groundwater viruses warrants further attention.

To explore host–virus matches further, linkages were refined to retain only a single predicted host (identified with the most robust prediction method), resulting in 100 linkages and 41 putative hosts spanning 16 phyla (Fig. 3A). Members of the *Pseudomonadota* were the most frequently predicted hosts (31.4% of linkages), followed by *Patescibacteria* (17.1%), and *Methylomirabilota* (14.3%). These observations are broadly consistent with the relative abundance of these hosts overall (Fig. 3B), with *Pseudomonadota* and *Patescibacteria* being the 1st and 2nd most abundant phyla in the MAG dataset, respectively, whereas *Methylomirabilota* was 10th (out of 40; Fig. S5). Over half of these linked hosts and viruses (*n* = 58) had similar abundances in groundwater (based on normalized genome coverage). This is illustrated by regression analyses showing near 1:1 correlations for pairs overall (Fig. 3C), suggesting they may represent integrated prophage in the lysogenic state rather than actively replicating viruses. Accordingly, 70% of predicted hosts, and just 13% of paired vOTUs were observed to be transcriptionally active (Tables S4 and S5). Two of the five inferred jumbo phages (vir_14 and vir_15) were associated with *Methylomirabilota* bacterium nzgw243 based on oligonucleotide frequency similarity. This bacterium harbors one of the largest estimated genome sizes in our dataset (6.9 Mbp, including a putative 40 kb-long integrated prophage). Jumbo phages have been isolated from diverse environments, and are shown to-date to mostly infect Gram-negative bacteria [58], consistent with the predicted infection of *Methylomirabilota,* which is a Gram-negative lineage [82]. Hosts of the other three large viruses remain unknown, but they likely infect other prokaryotes with relatively large genome and cell sizes, or possibly eukaryotes (the size ranges of giant amoeba viruses and jumbo phages overlap [83]).

As indicated above, a small number of viruses linked to hosts were co-binned with the host genome (Fig. 3A) based on tetranucleotide frequency, GC content, and differential coverage information. These viruses most likely represent prophage, although only two, vir_399 (binned with *Patescibacteria* nzgw482) and vir_483 (binned with *Pseudomonadota* nzgw550) were identified within host contigs (∼22 kbp of host sequence on one end for vir_399, and ∼30 kbp and ∼45 kbp on either end for vir_483). Host-matches based viral sequences such as these, with evidence of integration into the host chromosome, are deemed more robust than co-binning alone [46]. Of these, *Pseudomonadota* nzgw550 (19 785 TPM, 17-fold estimated genome coverage) and vir_483 (3 TPM, 0.5-fold estimated genome coverage) were both transcriptionally active, albeit to widely differing degrees (Table S10). Transcript reads mapped to the integrated vir_483 phage suggest low-level expression of genes encoding phage replication initiation protein (RstA1) and bacteriophage coat protein B, along with genes of unknown function, and could indicate lysis in a portion of the population. The *Patescibacteria* phylum, along with being the second most frequent phylum to be described as putative hosts, had 11 co-binned viral genomes. In addition, one vOTU was linked to a *Nanoarchaeota* archaeon. Viruses linked to ultra-small prokaryotes have been described previously [70, 84]. However, it remains unclear how microorganisms with such small cell and genomes sizes (1 ± 0.4 Mbp [24]) are capable of supporting viral infection and replication, and as ultra-small prokaryotes are considered host-dependent themselves [85], what role viruses play in the three-way symbiotic relationship.

### Impact of environmental parameters on host-virus pairs and viral activity

To investigate the environmental factors influencing viral community composition across samples, we performed a distance-based redundancy analysis (dbRDA) using Bray–Curtis dissimilarities between samples and groundwater physicochemical parameters (Fig. 2A). Viral communities followed bacterial and archaeal community dissimilarity patterns along a dissolved oxygen (DO) concentration gradient, as expected given the high host specificity typically exhibited by viruses [86]. This suggests that groundwater viruses, including the 61% predicted to infect multiple hosts in this study (Table S6), predominantly infect a single host, or infect co-abundant hosts sharing similar environmental niche preferences. Overall, the measured physicochemical parameters explained 50.5% of the variance between viral communities (adjusted R-squared; Table S7). Results indicate that DO was a major driver of viral community structure, along with nitrate content (permutation test, *P* <0.05; Table S7). Environmental influences on viral community composition are expected to be indirectly driven by host preferences. Consequently, we assessed the impact of environmental factors on the identified host–virus pairs.

In total, 59 host and virus pairs were positively or negatively correlated with at least one of 19 measured environmental variables (1–15 variables per pair; e.g. Fig. 2C, Tables S8 and S9). The environmental factor with the most prevalent association with virus-host relationships was groundwater iron content, with almost half of all pairs (44 out of 100 included in this analysis) significantly and almost exclusively correlating positively with total groundwater iron concentrations (Pearson’s correlation coefficient >0.6, *P* <0.05; Fig. 2B). Phage hosts and the tails of tailed phages are both predicted to be major iron sinks [87]. Iron is a critical element in protein function and bacterial/archaeal physiology, particularly for 4Fe–4S cluster-containing proteins that are involved in a large number of conserved cellular processes [88]. These proteins were also frequently detected in the recovered viral genomes, and could partially explain the strong correlation between host/viral abundances and iron content in aquifers. Strong correlations with iron may also be explained by the Ferrojan Horse Hypothesis, which hypothesizes that host iron co-opted for phage tail construction, is later used to initiate infection through competitive binding to cell surface iron receptors [87].

**Figure 4:**
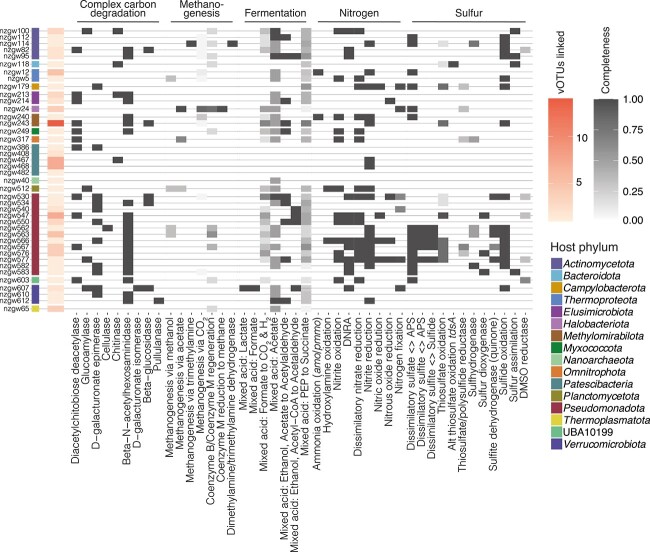
Biogeochemical cycling potential of putative prokaryotic hosts. Heatmap showing the completeness of metabolic pathways (columns) encoded by the putative hosts identified as most probable (colored by phylum; Table S6). KEGG KO of genes required for various metabolic and biosynthetic functions shown here were parsed using KEGG-decoder. Number of vOTUs linked to each host indicated in salmon color.

In addition to associations with iron, the normalized genome coverages of *Pseudomonadota*, *Planctomycetes*, *Verrucomicrobiota* and their respective viruses were strongly and positively correlated with DOC (e.g. Fig. 2C). These were among the strongest correlations overall (correlation coefficients 0.95 ± 0.03 SD). Members of these bacterial phyla are predicted to be involved in organic carbon cycling (complex carbon degradation, fermentation) in our groundwater samples (Fig. 4), and in other environments [89, 90]. Organic carbon is typically scarce in groundwater [91], to the extent that it has been reported to constrain microbial cell sizes [92]. Results here illustrate that this limiting resource—which is inferred to drive host abundance—similarly drives viral abundances.

**Figure 5:**
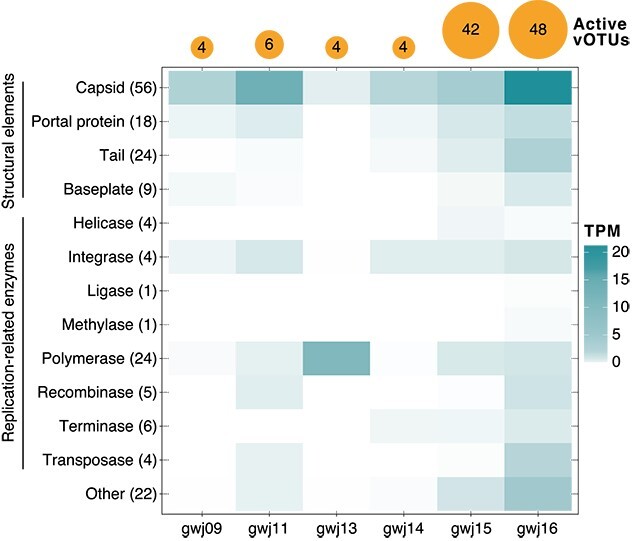
Expression of viral replication-related genes. Total TPM for genes involved in viral replication across samples (columns). Numbers in parentheses represent the total number of genes expressed for each category (rows). Orange bubbles on top show the total numbers of active vOTUs (log scaled).

**Figure 6:**
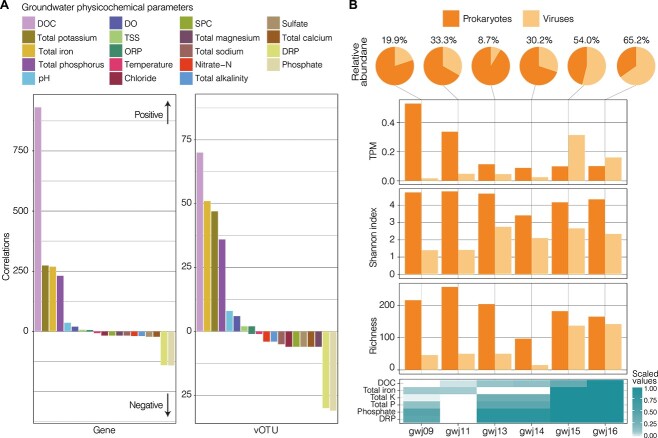
Impact of groundwater parameters on overall viral transcriptional activity. (A) Number of significant Pearson’s correlations (*P* <0.05) between groundwater physicochemical parameters and TPM values at the gene (left) and vOTU (right) levels. (B) Comparison between viral and prokaryotic community metrics. From top to bottom: Pie charts showing relative abundance (normalized genome coverages from metagenomic read mapping to MAGs and vOTUs); bar charts showing TPM (from metatranscriptomic read mapping to MAGs and vOTUs), Shannon index, and species richness (MAGs and vOTUs); and a heat map showing key groundwater physicochemical parameters scaled from 0 to 1.

Approximately half of host–virus pair relative abundances (normalized genome coverages) were poorly or inversely correlated with groundwater parameters (i.e. host and virus did not share the same correlations; Table S8), possibly due to differences between virus and host specific activities that are unaccounted for here. For example, either the phage life cycle (lytic versus lysogenic) [5] or host resistance to infection [93] could influence these correlations. However, of the 13 out of 100 host-associated vOTUs observed to be transcriptionally active (Table S10), expression was typically much lower than their respective host (0.26 to 19.93 fold lower, or 6.41 on average, based on transcripts per million after normalizing to genome size). As high viral transcription would be expected during lysis [5], this suggests low levels of viral production in the collected groundwater samples, whether due to environmental conditions at the time of sampling not suiting viral replication, viral replication in a subset of cells in host populations, or prophage being “grounded” and unable to undergo induction [94]. Regardless, viral transcriptional activity was evident in each of the groundwater samples (considering all vOTUs, between four and 48 exhibited activity per sample, and 72 [15%] were active overall; Fig. 5, Table S11). In addition, we identified the expression of replication-related genes (e.g. polymerase, tail, head and capsid genes; Fig. 5, Table S11) indicative of the lytic cycle. Correlations between groundwater parameters and overall viral transcription showed similar patterns at the gene and vOTU level (Fig. 6A). A large number of positive correlations were found between transcriptionally active viral genes/vOTUs and DOC, potassium, iron, and total phosphorus, whereas negative correlations were found between viral transcription and phosphate and DRP (Fig. 6A, Table S12). These findings therefore mirror some of the strongest or most prolific host-virus correlations identified (as discussed above), and suggest an association between viral lysis and higher DOC in groundwater (alongside higher viral relative abundance and alpha diversity; Fig. 6B). This may reflect an association between increased microbial biomass and lysis at higher DOC concentrations, and the recycling of this biomass into newly available DOC for microbial uptake via the viral shunt [95]. The association between resource availability in aquifers, microbial cell densities, and viral transcription thus warrants further exploration.

### Viruses are associated with microbial drivers of biogeochemical cycling of carbon, nitrogen, and sulfur in aquifers

To expand on the above observations, we investigated the potential for groundwater viruses to affect ecosystem function by infecting microbial drivers of biogeochemical cycles in aquifers. Microorganisms are the dominant form of cellular life in aquifers and play a critical role in transforming organic and inorganic compounds [96]. Viruses have the capacity to control host metabolism, and thereby biogeochemical cycles, via cell mortality, and subsequently community composition in a given ecosystem [2]. For all viruses linked to a putative host, we assessed the role of the host in carbon, sulfur, and nitrogen biogeochemical cycles in the groundwater samples (Fig. 4). Carbon, sulfur, and nitrogen are regarded to be key elements supporting microbial metabolism in the terrestrial subsurface [96]. Our dataset includes viruses putatively infecting bacteria and archaea involved in a wide range of biogeochemical processes related to the transformation of these elements.

Based on host-virus linkage associations, all pathways involved in the canonical groundwater nitrogen cycle [97] appeared to be impacted by viral infection, from ammonia oxidation (genomic potential found in *Thermoproteota* nzgw12 and *Methylomirabilota* nzgw240) to denitrification (genomic potential found in phylogenetically diverse microorganisms) (Fig. 4). For example, eight representatives of *Burkholderiales*, which appear to be important for nitrate reduction/complete denitrification and sulfur oxidation in these aquifers (MAGs nzgw547-nzgw577, Fig. 4), were linked to one to seven viruses each. In addition, among the predicted hosts were taxa involved in methane cycling, such as the *Methanoperedens* archaeon nzgw24, which is capable of reverse methanogenesis via the methyl-coenzyme M reductase (*mcrABCDG* genes) and F420-dependent 5,10-methenyltetrahydromethanopterin reductase (Mer) [98] (Fig. 4). Moreover, many viruses of heterotrophic microorganisms capable of degrading complex carbon or fermentation were identified (94 vOTUs). The metabolic products of which, such as lactate and acetate, can fuel sulfur and nitrogen cycling [99], demonstrating the potential for viruses to impact multiple steps in the microbial food chain. Altogether, these results suggest that viruses have the potential to indirectly affect microbial carbon, nitrogen, and sulfur cyclers in aquifers. The host organisms with these functions comprised between 3.2% and 36.8% of the total prokaryotic communities based on normalized genome coverage data (summed across the 16 groundwater samples), and between 12.0% and 40.2% of the prokaryotic transcriptional activity overall (Fig. S6). Although phage here are predicted to mediate turn-over of abundant biogeochemical cycle contributors, the high functional redundancy that is characteristic of aquifer systems [96] could diminish the impact on ecosystem function.

### Role of virally encoded auxiliary metabolic genes

In addition to their physical impact on microbial communities, phage can carry host-derived AMGs, which are used to manipulate the host cell metabolism during infection [4]. Due to strong selective pressure, it is believed that only AMGs that are maintaining and/or increasing fitness of the virus, or of its host, persist in genome sequences [1]. Using DRAM-v and VIBRANT, we predicted 205 AMGs from 101 vOTUs (21.6% of all vOTUs), including between 1 and 11 AMGs for the larger viral genomes (viral genomes >300 kbp; Tables S2 and S13).

**Figure 7:**
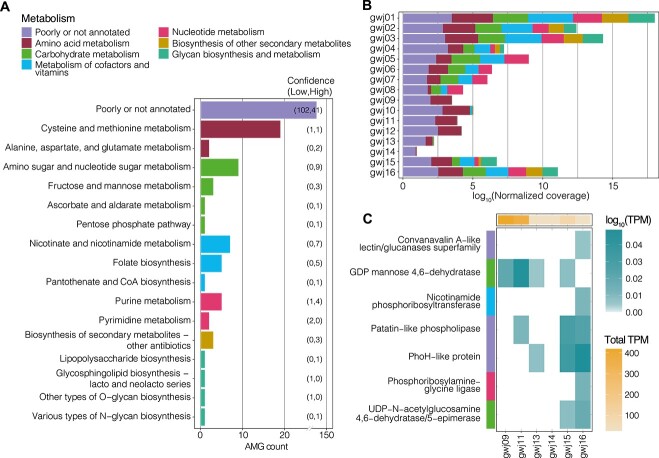
Virus-encoded AMG repertoire and expression. (A) Number AMGs identified in the recovered viral genomes based on KEGG pathways. Bars are colored based on KEGG metabolic categories. (B) Relative abundance of the identified AMGs across all 16 groundwater samples (based on vOTU normalized contig coverage). (C) Total TPM (all viral gene expression) across samples (top), and expression of AMGs shown as TPM values per AMG (bottom). Predicted AMGs expressed were, as shown from top to bottom: vir_472_43, vir_15_120, vir_20_197, vir_323_17, vir_146_43, vir_253_14, vir_243_51. Associated DO contents were 6.83 mg/L for sample gwj09 and 7.5 mg/l for gwj11 (oxic groundwater), and 1.06 mg/l for gwj13–14 and 0.37 mg/l for gwj15–16 (dysoxic). Samples with odd numbers are groundwater; those with even numbers (e.g. gwj16) are biomass-enriched groundwater (collected post down-well sonication to release sediment and biofilms into groundwater).

**Figure 8:**
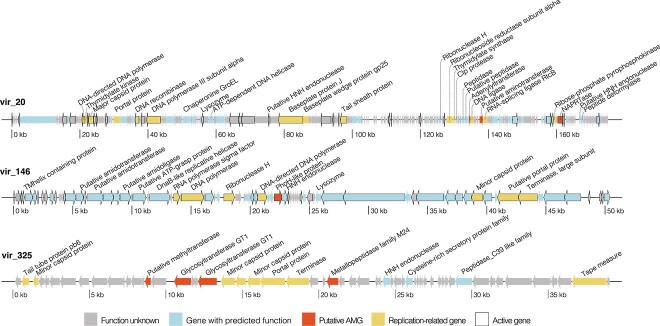
Examples of groundwater viral genomes harboring AMGs. Viral genomes selected illustrate a variety of AMG functions, and transcriptionally active AMGs. The linear genome maps indicate the location of putative AMGs, viral replication-related genes, and transcriptionally active genes (black outlines in top two maps).

Functional annotations of AMGs show that groundwater viruses acquired genes from a wide variety of cellular metabolic functions, spanning over 16 pathways defined by KEGG. Viruses predominantly encoded AMGs for amino acid metabolism (e.g. S-adenosylmethionine synthetase, methyltransferase, aminotransferase, asparagine synthase), followed by carbohydrate (primarily metabolism of various nucleotide sugars, or glycans, as below), and cofactor and vitamin metabolism (e.g. coenzyme-A biosynthesis, coenzyme nicotinamide adenine dinucleotide synthesis from vitamin B3, and folate biosynthesis intermediates) (Fig. 7A, vir_20 in Fig. 8, Table S13). Viruses with these AMGs were found across all sites (Fig. 7B). AMGs involved in cysteine and methionine biosynthesis (primarily DNA [cytosine-5]-methyltransferase 1, *dmc*), and nucleotide sugar biosynthesis (e.g. GDP-mannose 4,6 dehydratase) were overrepresented in groundwater viral genomes compared to other metabolisms (Fig. 7A, Table S13). These metabolic functions are reported to be enriched in marine viruses [100], pointing to consistent selective pressures for AMG acquisition across habitats. In some cases, these genes compensate for host metabolism, by filling metabolic gaps or by providing novel pathways as described in marine environments [101]. However, it is also thought that phages carry these genes to modulate host metabolism and redirect it to provide resources [5], and/or to recycle nucleotides from the cellular pool [102], for viral replication. They may also protect phage against host defenses. DNA methyltransferases, acquired from past or present hosts, are thought to serve a variety of functions, including avoidance of host restriction enzymes [103].

Of the 205 AMGs detected, 28 matched functions from the CAZy (Carbohydrate-Active enZYmes) database [104], and an additional 21 AMGs had predicted CAZy-related domains (Table S13). Most of the 49 virally encoded CAZymes were glycosyl transferases (biosynthesis of glycans; e.g. vir_325 in Fig. 8), and four were glycosyl hydrolases (catabolism). Three family 9 glycosyltransferase genes (GT9, lipopolysaccharide N-acetylglucosaminyltransferase) were predicted with high confidence in three different phages. This CAZyme participates in bacterial outer membrane (lipopolysaccharide) biogenesis [105]. Its presence in groundwater phage genomes, also described as AMGs in roseophage and other phage [106, 107], suggests LPS could be required for an efficient infection through host membrane disruption [107], or by preventing other viruses from entering the host cell via the same mechanism (i.e. targeting LPS). With close to one quarter of all putative AMGs detected in this study being involved in carbohydrate metabolism through CAZyme functions, results highlight direct and indirect roles for viruses in groundwater carbon cycling.

Overall, the vast majority of virally encoded AMGs in this study were involved in biosynthesis and transformation processes (e.g. lipopolysaccharide biosynthesis, and cofactor and vitamin biosynthesis), rather than catabolism of molecules in groundwater communities [108]. Although some AMGs encode for energy generating processes [9, 11, 13], anabolism (e.g. for nucleotide synthesis), metabolite interconversions, replication, and repair are in general common AMG traits [5, 88]. In addition, we found no genes involved in uptake of external resources, such as transporters or extracellular substrate binding proteins (aside from a gene encoding a potassium-transporting ATPase), as previously identified in marine viruses [6, 11]. We therefore speculate that groundwater viruses mostly rely on their host’s existing intracellular resources, given the scarcity of resources in aquifer environments.

We observed some functional redundancy between virally encoded AMGs and host metabolism. When comparing predicted AMG sequences to the gene content of respective hosts, 16 out of 205 AMGs showed homology to host genes (>30% amino acid identity over 50% length of viral query sequence), including 11 sharing 100% identity (Table S14). For example, we found that one putative glycosyltransferase and one nucleotide sugar dehydratase detected in vir_325 were homologous to genes found in its putative host *Halobacteriota* nzgw24 (100% protein sequence identity; Table S14). This strategy of duplicating host function could be employed by viruses to overcome the metabolic bottleneck of resource biosynthesis during viral reproduction in host cells. The remaining AMGs showed distant homology or no homology to host genes, suggesting that the majority of AMGs have diverged in their function over time, and/or that they introduce new functions to the host metabolic repertoire [101].

Although viral communities were active across all groundwater samples (Fig. S7), only seven AMGs were found to be expressed, mostly in sample gwj16—a sediment/biomass-enriched sample from dysoxic groundwater [24], where the viral community was also most active overall (Fig. 7C, Table S13). Of the expressed AMGs, two were involved in carbohydrate metabolism (amino and nucleotide sugar metabolism, and fructose and mannose metabolism), which is consistent with the prevalence of this metabolism in the groundwater virus AMG repertoire. The most expressed AMG was found in vir_146 (Fig. 7C), which also actively transcribed most of its other genes, including those associated with viral replication (Fig. 8). The AMG expressed by vir_146 encoded a PhoH-like protein (also detected in five other viruses but not expressed). PhoH is a commonly detected AMG that has been proposed as a viral biomarker in marine environments [109]. Its precise function remains poorly characterized, but is believed to be involved in the regulation of phosphorus under limiting conditions. Although expressed in the most phosphorus-rich groundwater samples (gwj15–16) in this study, this groundwater was oligotrophic and phosphorus-limited (concentrations were two orders of magnitude below eutrophication risk values for groundwater [110]). Such conditions could have triggered a phosphorus-related stress response in infected hosts.

## Conclusion

Little is known about the diversity and ecological role of viruses in aquifers. We identified over 400 novel groundwater viruses (vOTU clusters) across 16 groundwater samples. Although a small number were predicted based on genus-level clustering to belong to the dsDNA tailed bacteriophage *Caudoviricetes* class, most of them likely represent viral genera with no cultured representatives. Our results show that site-specific viral community structure was impacted indirectly by groundwater conditions, especially by differences in dissolved oxygen and nitrate, and that many host-virus pairs were strongly associated with DOC and total iron contents. By reconstructing prokaryotic MAGs and identifying viral contigs from the same metagenomic dataset, we predicted host–virus relationships *in silico* for a significant portion of vOTUs, and identified putative integrated prophage. Results show 104 groundwater viruses were associated with 48 microbial drivers of biogeochemical cycling in aquifers. Furthermore, 111 groundwater viruses were also found to carry a diverse repertoire of auxiliary metabolic genes. These genes were mostly involved in biosynthesis of important resources for viral replication. This suggests most AMGs acquired have a primary function unassociated with host survival—a feature of lytic rather than temperate viruses [111]. This potentially reflects an enrichment of AMGs in the lytic versus temperate viral fraction, as the temperate fraction is likely to be favored under the resource limiting conditions [3]. The presence and transcription of *phoH* may, nonetheless, contribute to host phosphorus regulation, although the implications for host survival are undetermined. Overall, we observed that at least 15% of groundwater vOTUs, and seven AMGs, mostly associated with carbohydrate metabolism, were transcriptionally active. Results provide insights into the underexplored diversity, varied prokaryotic host-associations of DNA viruses, and the narrow ecological niches shared by these viruses and their hosts in aquifers.

## Conflicts of interest

The authors declare no competing interests.

## Funding

Funding was provided by a MBIE Smart Ideas grant awarded to K.M. Handley (project UOAX1720), and we acknowledge support provided by Genomics Aotearoa for M. Hoggard (project 2101). We thank P. Abraham (ESR) for assistance in sampling and D. Waite and J.S. Boey for help with bioinformatics. Computational resources were provided by New Zealand eScience Infrastructure.

## Data availability

Sequence data were deposited with NCBI under BioProject PRJNA699054.

## Supplementary Material

Supplementary_Materials_Gios_2024_wrae035

Supplementary_Tables_Gios_2024_wrae035

## References

[1] Breitbart M , Bonnain C, Malki K. et al. Phage puppet masters of the marine microbial realm. Nat Microbiol 2018;3:754–66. 10.1038/s41564-018-0166-y. 29867096

[2] Suttle CA . The significance of viruses to mortality in aquatic microbial communities. Microb Ecol 1994;28:237–43. 10.1007/BF00166813. 24186450

[3] Brum JR , Hurwitz BL, Schofield O. et al. Seasonal time bombs: dominant temperate viruses affect Southern Ocean microbial dynamics. ISME J 2016;10:437–49. 10.1038/ismej.2015.125. 26296067 PMC4737935

[4] Breitbart M , Thompson LR, Suttle CA. et al. Exploring the vast diversity of marine viruses. Oceanography 2007;20:135–9. 10.5670/oceanog.2007.58.

[5] Thompson LR , Zeng Q, Kelly L. et al. Phage auxiliary metabolic genes and the redirection of cyanobacterial host carbon metabolism. Proc Natl Acad Sci USA 2011;108:E757–64. 10.1073/pnas.1102164108. 21844365 PMC3182688

[6] Warwick-Dugdale J , Buchholz HH, Allen MJ. et al. Host-hijacking and planktonic piracy: how phages command the microbial high seas. Virol J 2019;16:1–13. 10.1186/s12985-019-1120-1. 30709355 PMC6359870

[7] Mann NH , Cook A, Millard A. et al. Bacterial photosynthesis genes in a virus. Nature 2003;424:741. 10.1038/424741a. 12917674

[8] Millard A , Clokie MRJ, Shub DA. et al. Genetic organization of the *psbAD* region in phages infecting marine *Synechococcus* strains. Proc Natl Acad Sci USA 2004;101:11007–12. 10.1073/pnas.0401478101. 15263091 PMC503734

[9] Lindell D , Jaffe JD, Johnson ZI. et al. Photosynthesis genes in marine viruses yield proteins during host infection. Nature 2005;438:86–9. 10.1038/nature04111. 16222247

[10] Chen F , Lu J. Genomic sequence and evolution of marine cyanophage P60: a new insight on lytic and lysogenic phages. Appl Environ Microbiol 2002;68:2589–94. 10.1128/AEM.68.5.2589-2594.2002. 11976141 PMC127578

[11] Roux S , Brum JR, Dutilh BE. et al. Ecogenomics and potential biogeochemical impacts of globally abundant ocean viruses. Nature 2016;537:689–93. 10.1038/nature19366. 27654921

[12] Rohwer F , Segall A, Steward G. et al. The complete genomic sequence of the marine phage Roseophage SIO1 shares homology with nonmarine phages. Limnol Oceanogr 2000;45:408–18. 10.4319/lo.2000.45.2.0408.

[13] Kieft K , Zhou Z, Anderson RE. et al. Ecology of inorganic sulfur auxiliary metabolism in widespread bacteriophages. Nat Commun 2021;12:1–16. 10.1038/s41467-021-23698-5. 34108477 PMC8190135

[14] Chen LX , Méheust R, Crits-Christoph A. et al. Large freshwater phages with the potential to augment aerobic methane oxidation. Nat Microbiol 2020;5:1504–15. 10.1038/s41564-020-0779-9. 32839536 PMC7674155

[15] Holmfeldt K , Nilsson E, Simone D. et al. The Fennoscandian shield deep terrestrial virosphere suggests slow motion ‘boom and burst’ cycles. Commun Biol 2021;4:307. 10.1038/s42003-021-01810-1. 33686191 PMC7940616

[16] Kothari A , Roux S, Zhang H. et al. Ecogenomics of groundwater phages suggests niche differentiation linked to specific environmental tolerance. mSystems 2021;6:e0053721. 10.1128/mSystems.00537-21. 34184913 PMC8269241

[17] Kallies R , Hölzer M, Toscan RB. et al. Evaluation of sequencing library preparation protocols for viral metagenomic analysis from pristine aquifer groundwaters. Viruses 2019;11:484. 10.3390/v11060484. 31141902 PMC6631259

[18] Overholt WA , Hölzer M, Geesink P. et al. Inclusion of Oxford Nanopore long reads improves all microbial and viral metagenome-assembled genomes from a complex aquifer system. Environ Microbiol 2020;22:4000–13. 10.1111/1462-2920.15186. 32761733

[19] Pan D , Nolan J, Williams KH. et al. Abundance and distribution of microbial cells and viruses in an alluvial aquifer. Front Microbiol 2017;8:1199. 10.3389/fmicb.2017.01199. 28744257 PMC5504356

[20] Magnabosco C , Lin L-H, Dong H. et al. The biomass and biodiversity of the continental subsurface. Nat Geosci 2018;11:707–17. 10.1038/s41561-018-0221-6.

[21] Castelle CJ , Hug LA, Wrighton KC. et al. Extraordinary phylogenetic diversity and metabolic versatility in aquifer sediment. Nat Commun 2013;4:2120. 10.1038/ncomms3120. 23979677 PMC3903129

[22] He C , Keren R, Whittaker M. et al. Genome-resolved metagenomics reveals site-specific diversity of episymbiotic CPR bacteria and DPANN archaea in groundwater ecosystems. Nat Microbiol 2021;6:354–65. 10.1038/s41564-020-00840-5. 33495623 PMC7906910

[23] Mosley OE , Gios E, Weaver L. et al. Metabolic diversity and aero-tolerance in anammox bacteria from geochemically distinct aquifers. mSystems 2022;7:e01255–21. 35191775 10.1128/msystems.01255-21 PMC8862662

[24] Gios E , Mosley OE, Weaver L. et al. Ultra-small bacteria and archaea exhibit genetic flexibility towards groundwater oxygen content, and adaptations for attached or planktonic lifestyles. ISME Commun 2023;3:13. 10.1038/s43705-023-00223-x. 36808147 PMC9938205

[25] Rice E , Baird R, Eaton AD. APHA Standard Methods for the Examination of Water and Wastewater Standard Methods for the Examination of Water and Wastewater, 23rd edn. Washington, DC: American Public Health Association, American Water Works Association, Water Environment Federation, 2017.

[26] Chaumeil P-A , Mussig AJ, Hugenholtz P. et al. GTDB-Tk: a toolkit to classify genomes with the genome taxonomy database. Bioinformatics 2020;36:1925–7. 10.1093/bioinformatics/btz848. PMC7703759 31730192

[27] Price MN , Dehal PS, Arkin AP. FastTree 2—approximately maximum-likelihood trees for large alignments. PLoS On*e* 2010;5:e9490. 10.1371/journal.pone.0009490. 20224823 PMC2835736

[28] Letunic I , Bork P. Interactive tree of life (iTOL): an online tool for phylogenetic tree display and annotation. Bioinformatics 2007;23:127–8. 10.1093/bioinformatics/btl529. 17050570

[29] Graham ED , Heidelberg JF, Tully BJ. Potential for primary productivity in a globally-distributed bacterial phototroph. ISME J 2018;12:1861–6. 10.1038/s41396-018-0091-3. 29523891 PMC6018677

[30] Aramaki T , Blanc-Mathieu R, Endo H. et al. KofamKOALA: KEGG Ortholog assignment based on profile HMM and adaptive score threshold. Bioinformatics 2020;36:2251–2. 10.1093/bioinformatics/btz859. 31742321 PMC7141845

[31] Guo J , Bolduc B, Zayed AA. et al. VirSorter2: a multi-classifier, expert-guided approach to detect diverse DNA and RNA viruses. Microbiome 2021;9:37. 10.1186/s40168-020-00990-y. 33522966 PMC7852108

[32] Kieft K , Zhou Z, Anantharaman K. VIBRANT: automated recovery, annotation and curation of microbial viruses, and evaluation of viral community function from genomic sequences. Microbiome 2020;8:90. 10.1186/s40168-020-00867-0. 32522236 PMC7288430

[33] Ren J , Song K, Deng C. et al. Identifying viruses from metagenomic data using deep learning. Quant Biol 2020;8:64–77. 10.1007/s40484-019-0187-4. 34084563 PMC8172088

[34] Wood DE , Lu J, Langmead B. Improved metagenomic analysis with Kraken 2. Genome Biol 2019;20:257. 10.1186/s13059-019-1891-0. 31779668 PMC6883579

[35] Roux S , Adriaenssens EM, Dutilh BE. et al. Minimum information about an uncultivated virus genome (MIUVIG). Nat Biotechnol 2019;37:29–37. 10.1038/nbt.4306. 30556814 PMC6871006

[36] Marçais G , Delcher AL, Phillippy AM. et al. MUMmer4: a fast and versatile genome alignment system. PLoS Comput Biol 2018;14:e1005944. 10.1371/journal.pcbi.1005944. 29373581 PMC5802927

[37] Nayfach S , Camargo AP, Schulz F. et al. CheckV assesses the quality and completeness of metagenome-assembled viral genomes. Nat Biotechnol 2021;39:578–85. 10.1038/s41587-020-00774-7. 33349699 PMC8116208

[38] Langmead B , Trapnell C, Pop M. et al. Ultrafast and memory-efficient alignment of short DNA sequences to the human genome. Genome Biol 2009;10:R25. 10.1186/gb-2009-10-3-r25. 19261174 PMC2690996

[39] Probst AJ , Ladd B, Jarett JK. et al. Differential depth distribution of microbial function and putative symbionts through sediment-hosted aquifers in the deep terrestrial subsurface. Nat Microbiol 2018;3:328–36. 10.1038/s41564-017-0098-y. 29379208 PMC6792436

[40] Hyatt D , Chen G-L, Locascio PF. et al. Prodigal: prokaryotic gene recognition and translation initiation site identification. BMC Bioinformatics 2010;11:119. 10.1186/1471-2105-11-119. 20211023 PMC2848648

[41] Bin Jang H , Bolduc B, Zablocki O. et al. Taxonomic assignment of uncultivated prokaryotic virus genomes is enabled by gene-sharing networks. Nat Biotechnol 2019;37:632–9. 10.1038/s41587-019-0100-8. 31061483

[42] Buchfink B , Xie C, Huson DH. Fast and sensitive protein alignment using DIAMOND. Nat Methods 2015;12:59–60. 10.1038/nmeth.3176. 25402007

[43] Enright AJ , Van Dongen S, Ouzounis CA. An efficient algorithm for large-scale detection of protein families. Nucleic Acids Res 2002;30:1575–84. 10.1093/nar/30.7.1575. 11917018 PMC101833

[44] Nepusz T , Yu H, Paccanaro A. Detecting overlapping protein complexes in protein-protein interaction networks. Nat Methods 2012;9:471–2. 10.1038/nmeth.1938. 22426491 PMC3543700

[45] Shannon P , Markiel A, Ozier O. et al. Cytoscape: a software environment for integrated models of biomolecular interaction networks. Genome Res 2003;13:2498–504. 10.1101/gr.1239303. 14597658 PMC403769

[46] Emerson JB , Roux S, Brum JR. et al. Host-linked soil viral ecology along a permafrost thaw gradient. Nat Microbiol 2018;3:870–80. 10.1038/s41564-018-0190-y. 30013236 PMC6786970

[47] Skennerton CT , Imelfort M, Tyson GW. Crass: identification and reconstruction of CRISPR from unassembled metagenomic data. Nucleic Acids Res 2013;41:e105. 10.1093/nar/gkt183. 23511966 PMC3664793

[48] Couvin D , Bernheim A, Toffano-Nioche C. et al. CRISPRCasFinder, an update of CRISRFinder, includes a portable version, enhanced performance and integrates search for CAS proteins. Nucleic Acids Res 2018;46:W246–51. 10.1093/nar/gky425. 29790974 PMC6030898

[49] Laslett D , Canback B. ARAGORN, a program to detect tRNA genes and tmRNA genes in nucleotide sequences. Nucleic Acids Res 2004;32:11–6. 10.1093/nar/gkh152. 14704338 PMC373265

[50] Ahlgren NA , Ren J, Lu YY. et al. Alignment-free $d_2^*$ oligonucleotide frequency dissimilarity measure improves prediction of hosts from metagenomically-derived viral sequences. Nucleic Acids Res 2017;45:39–53. 10.1093/nar/gkw1002. 27899557 PMC5224470

[51] Shaffer MJ , Borton MA, McGivern BB. et al. DRAM for distilling microbial metabolism to automate the curation of microbiome function. Nucleic Acids Res 2020;48:8883–900. 10.1093/nar/gkaa621. 32766782 PMC7498326

[52] Kopylova E , Noé L, Touzet H. SortMeRNA: fast and accurate filtering of ribosomal RNAs in metatranscriptomic data. Bioinformatics 2012;28:3211–7. 10.1093/bioinformatics/bts611. 23071270

[53] Langmead B , Salzberg SL. Fast gapped-read alignment with Bowtie 2. Nat Methods 2012;9:357–9. 10.1038/nmeth.1923. 22388286 PMC3322381

[54] Liao Y , Smyth GK, Shi W. featureCounts: an efficient general purpose program for assigning sequence reads to genomic features. Bioinformatics 2014;30:923–30. 10.1093/bioinformatics/btt656. 24227677

[55] R Core Team . R: A Language and Environment for Statistical Computing. Vienna, Austria: R Foundation for Statistical Computing, 2021, https://www.R-project.org.

[56] Oksanen J , Simpson GL, Blanchet GF. et al. Vegan: Community Ecology Package*.* R package version 2.5.6, 1–29 https://CRAN.R-project.org/package=vegan.

[57] Hewson I , Fuhrman J. Viriobenthos production and virioplankton sorptive scavenging by suspended sediment particles in coastal and pelagic waters. Microb Ecol 2003;46:337–47. 10.1007/s00248-002-1041-0. 14502409

[58] Yuan Y , Gao M. Jumbo bacteriophages: an overview. Front Microbiol 2017;8:403. 10.3389/fmicb.2017.00403. 28352259 PMC5348500

[59] Chaikeeratisak V , Nguyen K, Khanna K. et al. Assembly of a nucleus-like structure during viral replication in bacteria. Science 2017;355:194–7. 10.1126/science.aal2130. 28082593 PMC6028185

[60] Ackermann HW . 5500 Phages examined in the electron microscope. Arch Virol 2007;152:227–43. 10.1007/s00705-006-0849-1. 17051420

[61] Ackermann HW , Prangishvili D. Prokaryote viruses studied by electron microscopy. Arch Virol 2012;57:1843–9. 10.1007/s00705-012-1383-y 22752841

[62] Gregory AC , Zayed AA, Conceição-Neto N. et al. Marine DNA viral macro- and microdiversity from pole to pole. Cell 2019;177:1109–1123.e14. 10.1016/j.cell.2019.03.040. 31031001 PMC6525058

[63] López-Pérez M , Haro-Moreno JM, de la Torre JR. et al. Novel *Caudovirales* associated with Marine Group I Thaumarchaeota assembled from metagenomes. Environ Microbiol 2019;21:1980–8. 10.1111/1462-2920.14462. 30370610

[64] Moon K , Jeon JH, Kang I. et al. Freshwater viral metagenome reveals novel and functional phage-borne antibiotic resistance genes. Microbiome 2020;8:75. 10.1186/s40168-020-00863-4. 32482165 PMC7265639

[65] Malki K , Rosario K, Sawaya NA. et al. Prokaryotic and viral community composition of Florida springs. Am Soc Microbiol 2020;11:e00436–20. 10.1128/mBio.00436-20 PMC7157768 32265327

[66] Costeira R , Doherty R, Allen CCR. et al. Analysis of viral and bacterial communities in groundwater associated with contaminated land. Sci Total Environ 2019;656:1413–26. 10.1016/j.scitotenv.2018.11.429. 30625669

[67] Kyle JE , Eydal HSC, Ferris FG. et al. Viruses in granitic groundwater from 69 to 450 m depth of the Äspö hard rock laboratory. Sweden ISME J 2008;2:571–4. 10.1038/ismej.2008.18. 18288217

[68] Hylling O , Carstens AB, Kot W. et al. Two novel bacteriophage genera from a groundwater reservoir highlight subsurface environments as underexplored biotopes in bacteriophage ecology. Sci Rep 2020;10:11879. 10.1038/s41598-020-68389-1. 32681144 PMC7368026

[69] Hug LA , Thomas BC, Brown CT. et al. Aquifer environment selects for microbial species cohorts in sediment and groundwater. ISME J 2015;9:1846–56. 10.1038/ismej.2015.2. 25647349 PMC4511941

[70] Li Z , Pan D, Wei G. et al. Deep sea sediments associated with cold seeps are a subsurface reservoir of viral diversity. ISME J 2021;15:2366–78. 10.1038/s41396-021-00932-y. 33649554 PMC8319345

[71] Holmfeldt K , Middelboe M, Nybroe O. et al. Large variabilities in host strain susceptibility and phage host range govern interactions between lytic marine phages and their *Flavobacterium* hosts. Appl Environ Microbiol 2007;73:6730–9. 10.1128/AEM.01399-07. 17766444 PMC2074958

[72] Hammerl JA , Göllner C, Al Dahouk S. et al. Analysis of the first temperate broad host range Brucellaphage (BiPBO1) isolated from *B. inopinata*. Front Microbiol 2016;7:24. 26858702 10.3389/fmicb.2016.00024 PMC4729917

[73] Peters DL , Lynch KH, Stothard P. et al. The isolation and characterization of two *Stenotrophomonas maltophilia* bacteriophages capable of cross-taxonomic order infectivity. BMC Genomics 2015;16:664. 10.1186/s12864-015-1848-y. 26335566 PMC4559383

[74] Roux S , Krupovic M, Daly RA. et al. Cryptic inoviruses revealed as pervasive in bacteria and archaea across Earth’s biomes. Nat Microbiol 2019;4:1895–906. 10.1038/s41564-019-0510-x. 31332386 PMC6813254

[75] Roux S , Hawley AK, Torres Beltran M. et al. Ecology and evolution of viruses infecting uncultivated SUP05 bacteria as revealed by single-cell- and meta-genomics. Elife 2014;3: e03125. 10.7554/eLife.03125. 25171894 PMC4164917

[76] Jensen EC , Schrader HS, Rieland B. et al. Prevalence of broad-host-range lytic bacteriophages of *Sphaerotilus natans*, *Escherichia coli*, and *Pseudomonas aeruginosa*. Appl Environ Microbiol 1998;64:575–80. 10.1128/AEM.64.2.575-580.1998. 9464396 PMC106085

[77] Buchholz HH , Michelsen ML, Bolaños LM. et al. Efficient dilution-to-extinction isolation of novel virus–host model systems for fastidious heterotrophic bacteria. ISME J 2021;15:1585–98. 10.1038/s41396-020-00872-z. 33495565 PMC8163748

[78] Malki K , Kula A, Bruder K. et al. Bacteriophages isolated from Lake Michigan demonstrate broad host-range across several bacterial phyla. Virol J 2015;12:164. 10.1186/s12985-015-0395-0. 26453042 PMC4600314

[79] Schwarzer D , Buettner FFR, Browning C. et al. A multivalent adsorption apparatus explains the broad host range of phage phi92: a comprehensive genomic and structural analysis. J Virol 2012;86:10384–98. 10.1128/JVI.00801-12. 22787233 PMC3457257

[80] de Jonge PA , Nobrega FL, Brouns SJJ. et al. Molecular and evolutionary determinants of bacteriophage host range. Trends Microbiol 2019;27:51–63. 10.1016/j.tim.2018.08.006. 30181062

[81] Paez-Espino D , Eloe-Fadrosh EA, Pavlopoulos GA. et al. Uncovering Earth’s virome. Nature 2016;536:425–30. 10.1038/nature19094. 27533034

[82] Wu ML , van Teeseling MCF, Willems MJR. et al. Ultrastructure of the denitrifying methanotroph “*Candidatus* Methylomirabilis oxyfera,” a novel polygon-shaped bacterium. J Bacteriol 2012;194:284–91. 10.1128/JB.05816-11. 22020652 PMC3256638

[83] Colson P , La Scola B, Raoult D. Giant viruses of amoebae: a journey through innovative research and paradigm changes. Annu Rev Virol 2017;4:61–85. 10.1146/annurev-virology-101416-041816. 28759330

[84] Munson-McGee JH , Rooney C, Young MJ. An uncultivated virus infecting a nanoarchaeal parasite in the hot springs of Yellowstone National Park. J Virol 2020;94:e01213–9. 31666377 10.1128/JVI.01213-19 PMC7000964

[85] Brown CT , Hug LA, Thomas BC. et al. Unusual biology across a group comprising more than 15% of domain bacteria. Nature 2015;523:208–11. 10.1038/nature14486. 26083755

[86] Ackermann HW , Audurier A, Berthiaume L. et al. Guidelines for bacteriophage characterization. Adv Virus Res 1978;23:1–24. 34986 10.1016/s0065-3527(08)60096-2

[87] Bonnain C, Breitbart M, Buck KN. The Ferrojan horse hypothesis: iron-virus interactions in the ocean. Front Mar Sci 2016;3:82.

[88] Hurwitz BL , Brum JR, Sullivan MB. Depth-stratified functional and taxonomic niche specialization in the ‘core’ and ‘flexible’ Pacific Ocean Virome. ISME J 2015;9:472–84. 10.1038/ismej.2014.143. 25093636 PMC4303639

[89] He S , Stevens SLR, Chan L-K. et al. Ecophysiology of freshwater Verrucomicrobia inferred from metagenome-assembled genomes. mSphere 2017;2:e00277–17. 28959738 10.1128/mSphere.00277-17 PMC5615132

[90] Zhou Z , Tran PQ, Kieft K. et al. Genome diversification in globally distributed novel marine Proteobacteria is linked to environmental adaptation. ISME J 2020;14:2060–77. 10.1038/s41396-020-0669-4. 32393808 PMC7367891

[91] Griebler C , Lueders T. Microbial biodiversity in groundwater ecosystems. Freshw Biol 2009;54:649–77. 10.1111/j.1365-2427.2008.02013.x.

[92] Bengtsson G . Growth and metabolic flexibility in groundwater bacteria. Microb Ecol 1989;18:235–48. 10.1007/BF02075811. 24196204

[93] Sharon I , Morowitz MJ, Thomas BC. et al. Time series community genomics analysis reveals rapid shifts in bacterial species, strains, and phage during infant gut colonization. Genome Res 2012;23:111–20. 10.1101/gr.142315.112. 22936250 PMC3530670

[94] Ramisetty BCM , Sudhakari PA. Bacterial ‘grounded’ prophages: hotspots for genetic renovation and innovation. Front Genet 2019;10:65. 10.3389/fgene.2019.00065. 30809245 PMC6379469

[95] Wilhelm SW , Suttle CA. Viruses and nutrient cycles in the sea: viruses play critical roles in the structure and function of aquatic food webs. Bioscience 1999;49:781–8. 10.2307/1313569.

[96] Anantharaman K , Brown CT, Hug LA. et al. Thousands of microbial genomes shed light on interconnected biogeochemical processes in an aquifer system. Nat Commun 2016;7:13219. 10.1038/ncomms13219. 27774985 PMC5079060

[97] Kuypers MMM , Marchant HK, Kartal B. The microbial nitrogen-cycling network. Nat Rev Microbiol 2018;16:263–76. 10.1038/nrmicro.2018.9. 29398704

[98] Haroon MF , Hu S, Shi Y. et al. Anaerobic oxidation of methane coupled to nitrate reduction in a novel archaeal lineage. Nature 2013;500:567–70. 10.1038/nature12375. 23892779

[99] Baker BJ , Lazar CS, Teske AP. et al. Genomic resolution of linkages in carbon, nitrogen, and sulfur cycling among widespread estuary sediment bacteria. Microbiome 2015;3:14. 10.1186/s40168-015-0077-6. 25922666 PMC4411801

[100] Enav H , Mandel-Gutfreund Y, Béjà O. Comparative metagenomic analyses reveal viral-induced shifts of host metabolism towards nucleotide biosynthesis. Microbiome 2014;2:9. 10.1186/2049-2618-2-9. 24666644 PMC4022391

[101] He T , Li H, Zhang X. Deep-sea hydrothermal vent viruses compensate for microbial metabolism in virus-host interactions. MBio 2017;8:e00893–17. 28698277 10.1128/mBio.00893-17 PMC5513705

[102] Lavigne R , Lecoutere E, Wagemans J. et al. A multifaceted study of *Pseudomonas aeruginosa* shutdown by virulent podovirus LUZ19. MBio 2013;4:e00061–13. 23512961 10.1128/mBio.00061-13 PMC3604761

[103] Murphy J , Mahoney J, Ainsworth S. et al. Bacteriophage orphan DNA methyltransferases: insights from their bacterial origin, function, and occurrence. Appl Environ Microbiol 2013;79:7547–55. 10.1128/AEM.02229-13. 24123737 PMC3837797

[104] Drula E , Garron M, Doğan S. et al. The carbohydrate-active enzyme database: functions and literature. Nucleic Acids Res 2021;50:D571–7. 10.1093/nar/gkab1045. PMC8728194 34850161

[105] Kadrmas JL , Raetz CRH. Enzymatic synthesis of lipopolysaccharide in *Escherichia coli*. J Biol Chem 1998;273:2799–807. 10.1074/jbc.273.5.2799. 9446588

[106] Huang X , Jiao N, Zhang R. The genomic content and context of auxiliary metabolic genes in roseophages. Environ Microbiol 2021;23:3743–57. 10.1111/1462-2920.15412. 33511765

[107] Mutalik VK , Adler BA, Rishi HS. et al. High-throughput mapping of the phage resistance landscape in *E. coli*. PLoS Biol 2020;18:e3000877. 10.1371/journal.pbio.3000877. 33048924 PMC7553319

[108] Cheng Z , Li X, Palomo A. et al. Virus impacted community adaptation in oligotrophic groundwater environment revealed by Hi-C coupled metagenomic and viromic study. J Hazard Mater 2023;458:131944. 10.1016/j.jhazmat.2023.131944. 37390685

[109] Goldsmith DB , Crosti G, Dwivedi B. et al. Development of PhoH as a novel signature gene for assessing marine phage diversity. Appl Environ Microbiol 2011;77:7730–9. 10.1128/AEM.05531-11. 21926220 PMC3209181

[110] Warrack J , Kang M, von Sperber C. Groundwater phosphorus concentrations: global trends and links with agricultural and oil and gas activities. Environ Res Lett 2021;17:014014. 10.1088/1748-9326/ac31ef.

[111] Luo XQ , Wang P, Li JL. et al. Viral community-wide auxiliary metabolic genes differ by lifestyles, habitats, and hosts. Microbiome 2022;10:190. 10.1186/s40168-022-01384-y. 36333738 PMC9636769

