# Supplementary material for: High niche specificity and host genetic diversity of groundwater viruses
Source: ISME J. 2024 Mar 7;18(1):wrae035. doi: 10.1093/ismejo/wrae035 (PMC10980836; doi:10.1093/ismejo/wrae035)
Supplement: Supplementary_Materials_Gios_2024_wrae035 [file supplementary_materials_gios_2024_wrae035.pdf]

Supplementary Materials for Gios *et al*. (methods, references, and figures)

SUPPLEMENTARY METHODS

Identification of auxiliary metabolic genes (AMGs).

VIBRANT [1] was modified to omit any filtering steps to solely generate annotations for all contigs as follows: *VIBRANT_run.py* was edited (line 138) to reduce the required open reading frame count to zero to exclude filtering by the absence of open reading frames. *VIBRANT_annotation.py* was edited to exclude filtering at each step in the process by overwriting the filtered fasta file with the original unfiltered fasta file (lines 423, 593), and excluding filtering by those IDs found in the variable 'keep' (line 751).

Taxonomic assignment for unassembled metagenomic reads

Protein coding sequences were predicted from quality trimmed metagenomic reads using prodigal v2.6.3 (-p meta) [2] and compared to the NCBI nr database (downloaded 22nd May 2019) using DIAMOND v0.9.22 [3]. MEGAN 6 (v6.18.11) [4] was used to parse DIAMOND blast results and domain-level taxonomy (i.e., Bacteria, Archaea, Eukaryotes, or to Viruses) was assigned using the naïve LCA Algorithm with default parameters (min score=50, max expected=0.01, top percent=10, min support percent=0.05).

SUPPLEMENTARY REFERENCES

1. Kieft K, Zhou Z, Anantharaman K. VIBRANT: automated recovery, annotation and curation of microbial viruses, and evaluation of viral community function from genomic sequences. Microbiome. 2020;8:90.

2. Hyatt D, Chen G-L, Locascio PF, Land ML, Larimer FW, Hauser LJ. Prodigal: prokaryotic gene recognition and translation initiation site identification. BMC Bioinformatics. 2010;11:119.

3. Buchfink B, Xie C, Huson DH. Fast and sensitive protein alignment using DIAMOND. Nat Methods. 2015;12:59–60.

4. Huson DH, Auch AF, Qi J, Schuster SC. MEGAN analysis of metagenomic data. Genome Res. 2007;17:377–86.

SUPPLEMENTARY FIGURES

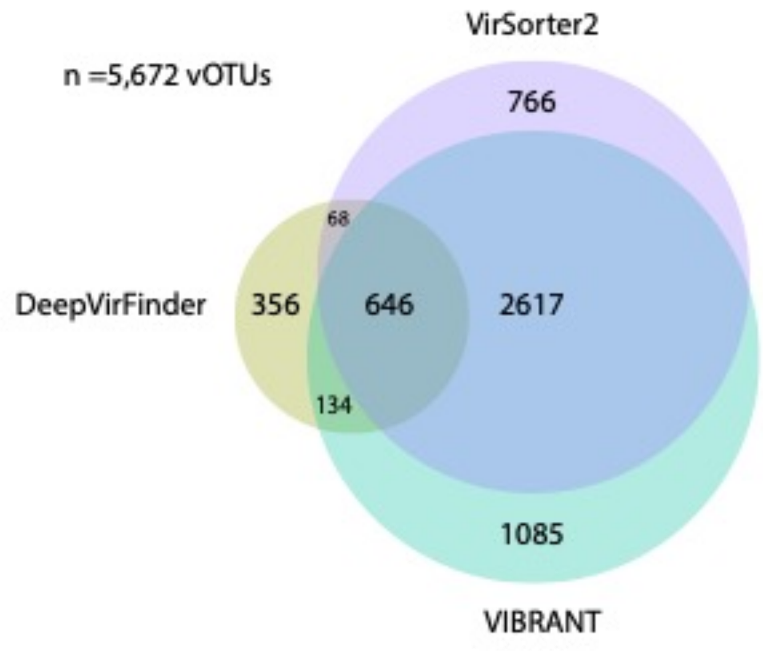

Figure S1. Venn diagram showing the number of vOTUs supported by each method (i.e., viral contigs detected in association with a vOTU). Across methods a total of 12,990 viral genomes clustered into 5,672 non quality filtered vOTUs, with 3,465 vOTUs detected by at least two methods, and most detected jointly by VIBRANT and VirSorter2 (n = 3,263). From this initial set of vOTUs, 483 were assessed as high-quality based on CheckV, and 468 after manual filtering based on criteria mentioned in Methods section.

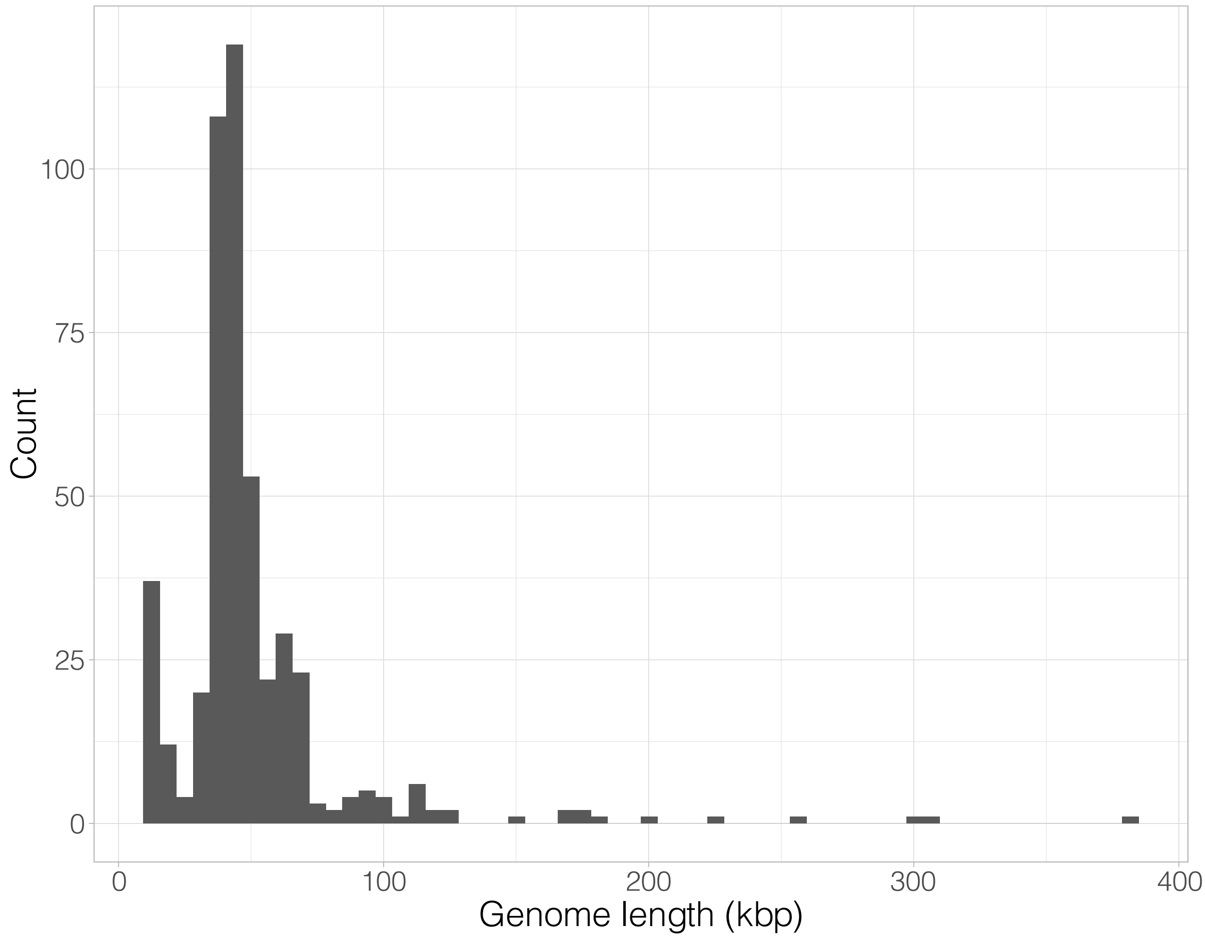

Figure S2. Genome length distribution of groundwater vOTU representative sequences recovered in this study.

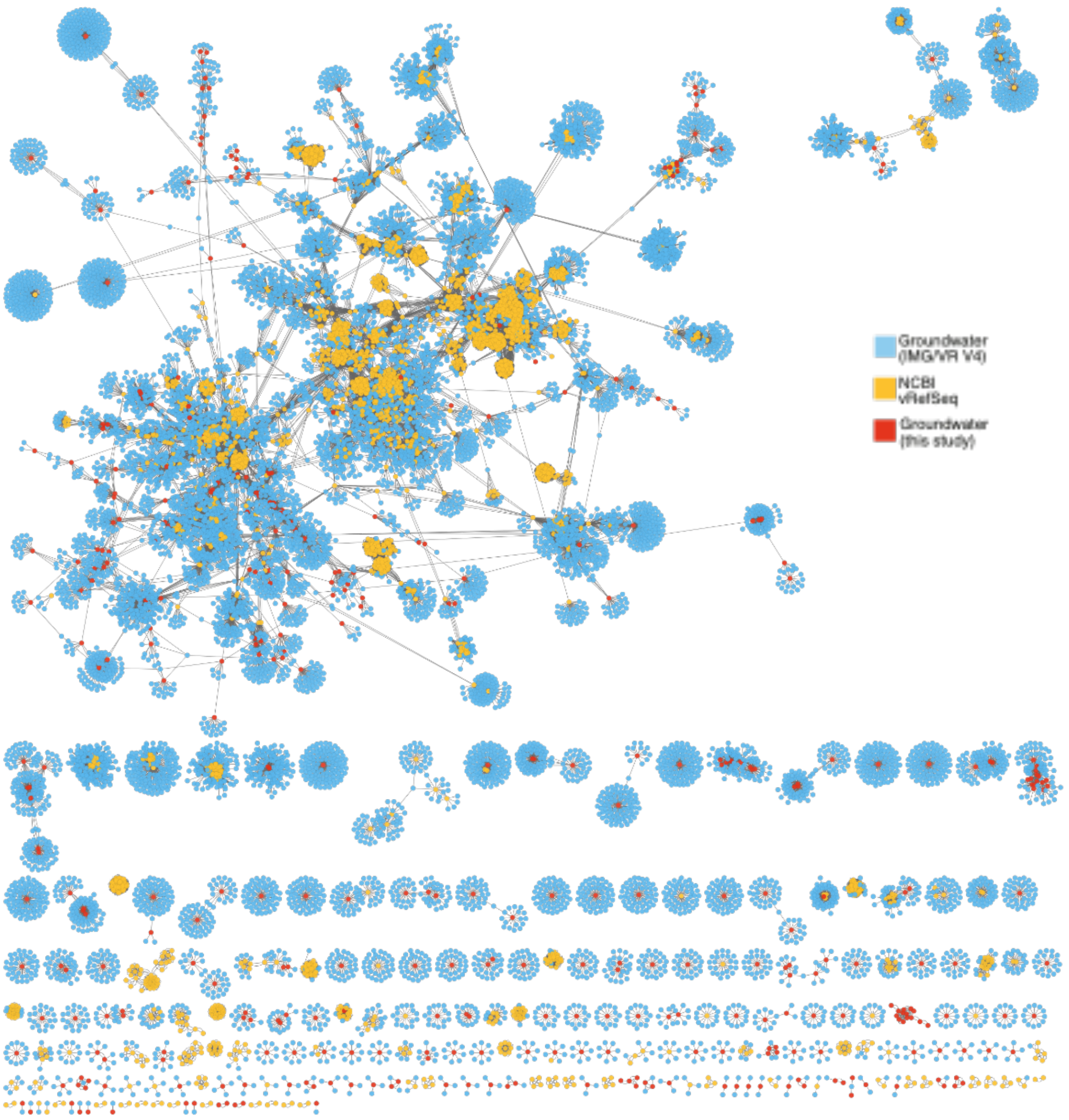

Figure S3. Network of shared predicted protein content between high-quality groundwater vOTUs (n = 468) (red), NCBI vRefSeq sequences v201 (n = 3,502) (yellow), and groundwater viral sequences from IMG/VR v4 (n = 160,465) (blue). For visualisation following clustering, IMG/VR viruses that did not share an edge with vOTU or vRefSeq genomes were removed. Viral genomes are represented by the nodes (ovals). Shared edges (grey lines) indicate shared protein content.

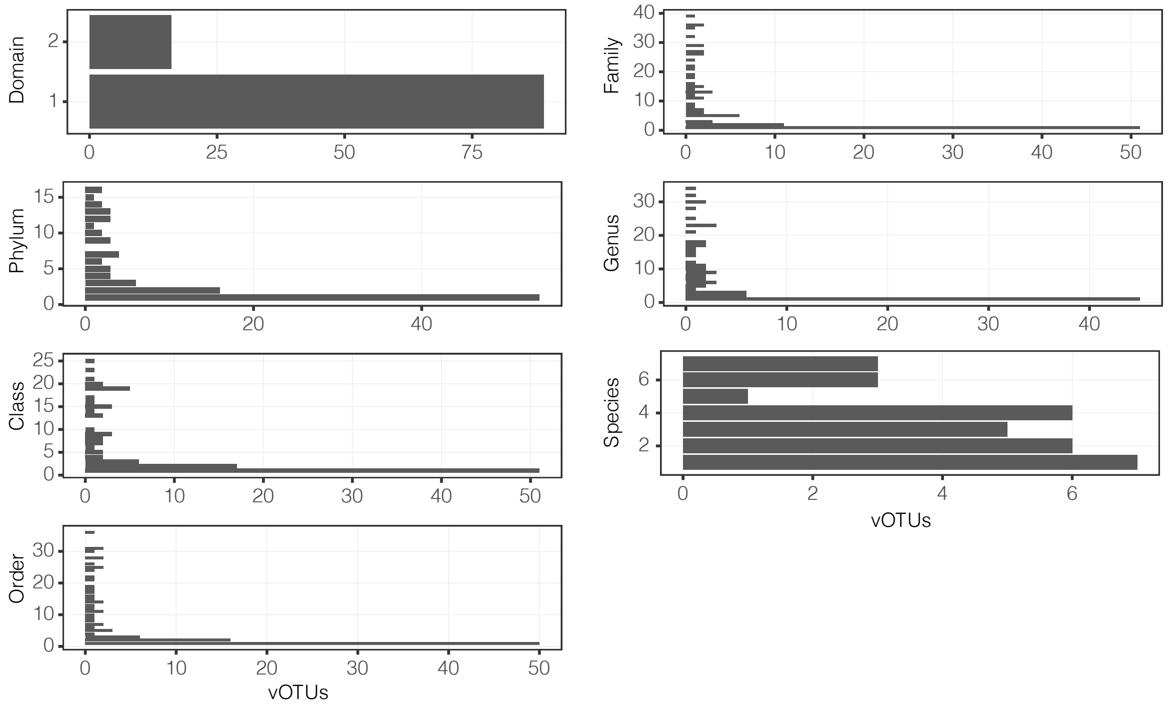

Figure S4. Number of vOTUs linked to hosts (MAGs) at each taxonomic level (Table S5). Only levels with known taxonomy were considered. The species plot excludes vOTUs with no species level matches (n=104). At every taxonomic level results show a greater frequency of higher specificity matches.

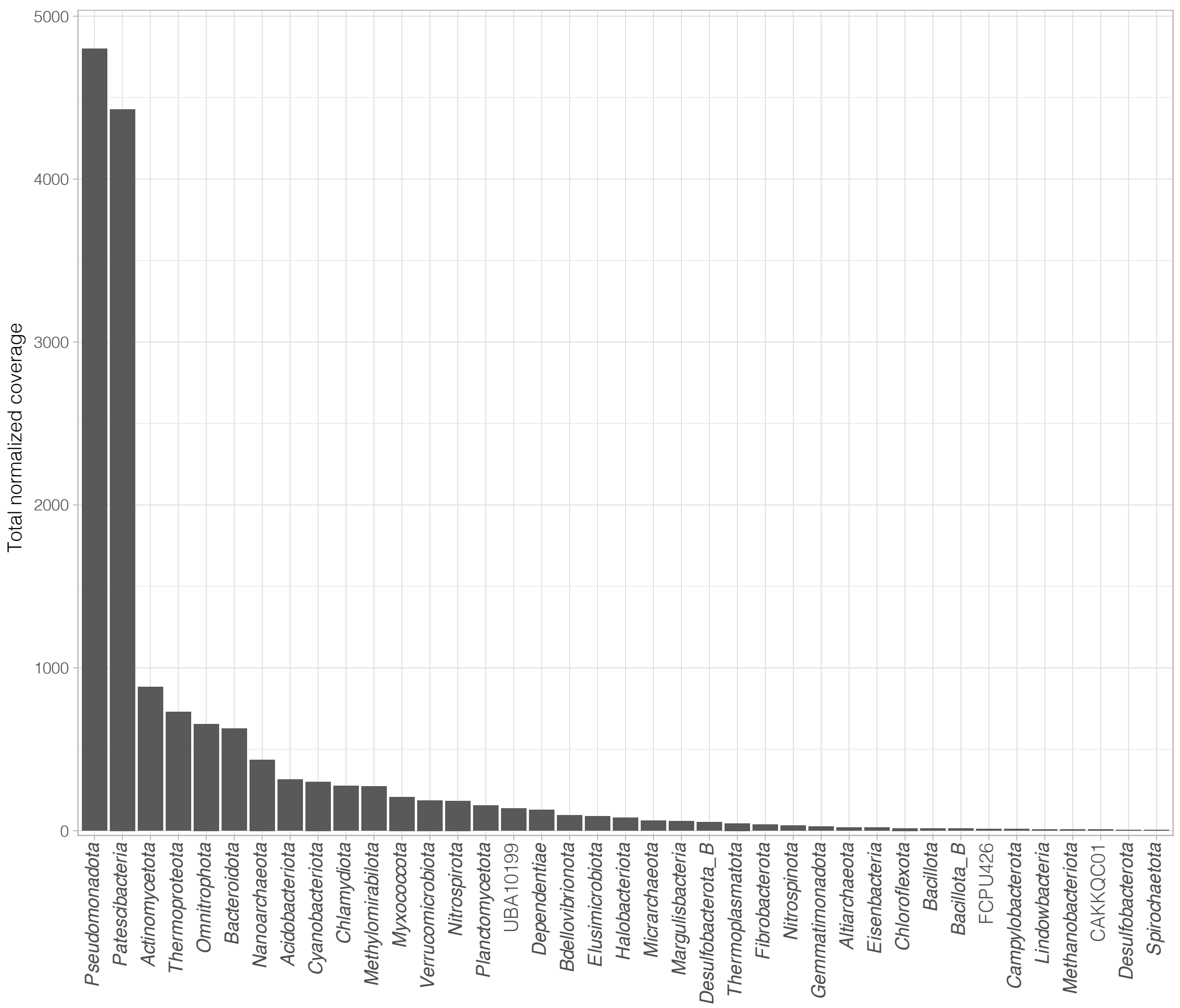

Figure S5. Rank abundance plot showing the total relative abundance of 396 prokaryotic MAGs across 16 groundwater samples. Abundances were summed across sites and by phyla.

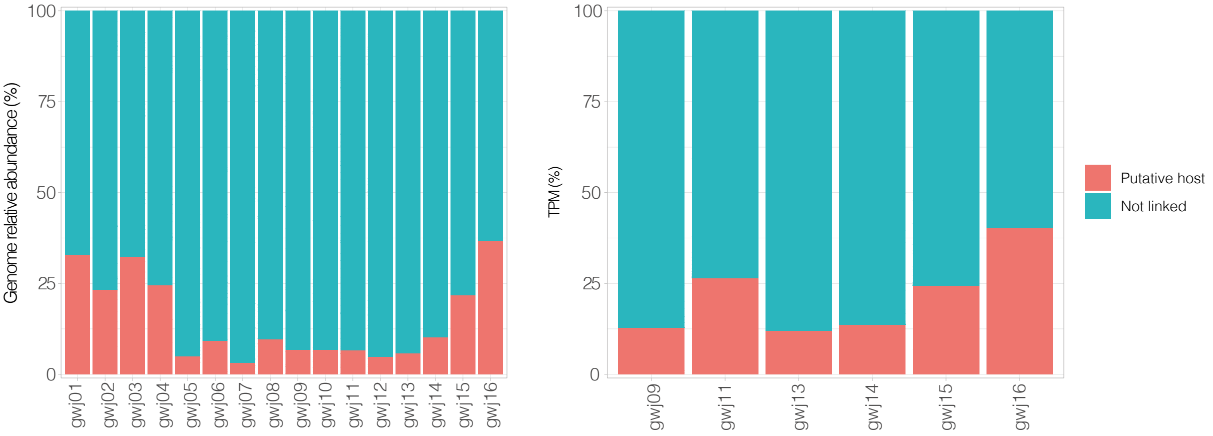

Figure S6. Prokaryotic genome relative abundance (left) and proportion of total TPM expressed by putative viral prokaryote hosts (right).

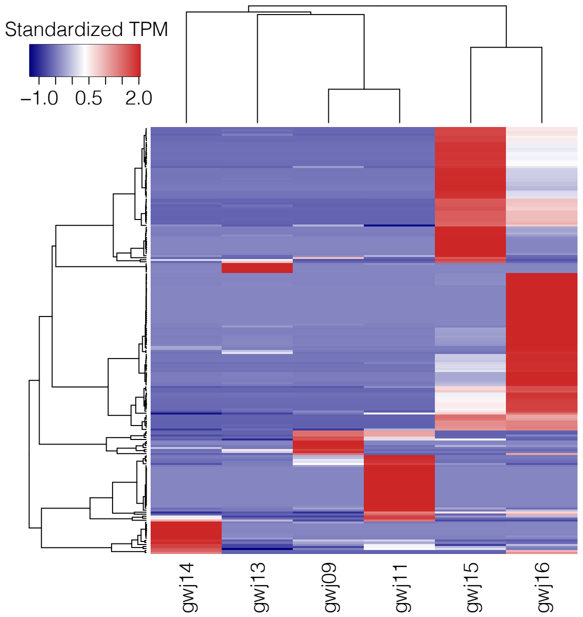

Figure S7. Heatmap showing overall gene expression of viral communities across six groundwater samples, with rows representing vOTUs (no labels shown), and columns representing samples. Dendrograms represent ward.D2 method clustering.
